# Convenient syntheses of 2-acylamino-4-halothiazoles and acylated derivatives using a versatile Boc-intermediate†

**DOI:** 10.1039/d4ra04959d

**Published:** 2024-09-02

**Authors:** Sophie Pate, Joshua Taujanskas, Robyn Wells, Craig M. Robertson, Paul M. O'Neill, Andrew V. Stachulski

**Affiliations:** a Department of Chemistry, University of Liverpool Liverpool L69 7ZD UK stachuls@liv.ac.uk +44-(0)151-794-3482 +44-(0)151-794-3482

## Abstract

The 2-aminothiazole grouping is a significant feature of many series of biologically active molecules, including antibiotics, anticancer agents and NSAIDs. We have a longstanding interest in the synthesis and biological evaluation of thiazolides, *viz.* [2-hydroxyaroyl-*N*-(thiazol-2-yl)-amides] which have broad spectrum antiinfective, especially antiviral, properties. However, 2-amino-4-substituted thiazoles, especially 4-halo examples, are not easily available. We now report practical, efficient syntheses of this class from readily available pseudothiohydantoin, or 2-aminothiazol-4(5*H*)-one: the key intermediate was its Boc derivative, from which, under Appel-related conditions, Br, Cl and I could all be introduced at C(4). Whereas 2-amino-4-Br/4-Cl thiazoles gave low yields of mixed products on acylation, including a bis-acyl product, further acylation of the Boc intermediates, with a final mild deprotection step, afforded the desired thiazolides cleanly and in good yields. In contrast, even mild hydrolysis of 2-acetamido-4-chlorothiazole led to decomposition with fast reversion to 2-aminothiazol-4(5*H*)-one. We also present a correction of a claimed synthesis of 2-acetamido-4-chlorothiazole, which in fact produces its 5-chloro isomer.

## Abstract 2

Pseudothiohydantoin was readily converted into 2-amino-4-halothiazoles in Boc-protected form. The products were readily converted into potential antiviral thiazolides.

## Graphical abstract

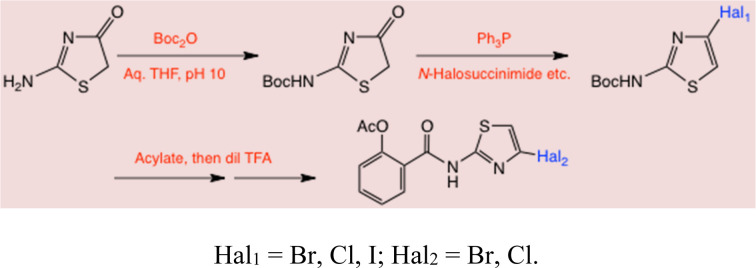

## Introduction

**Fig. 1:**
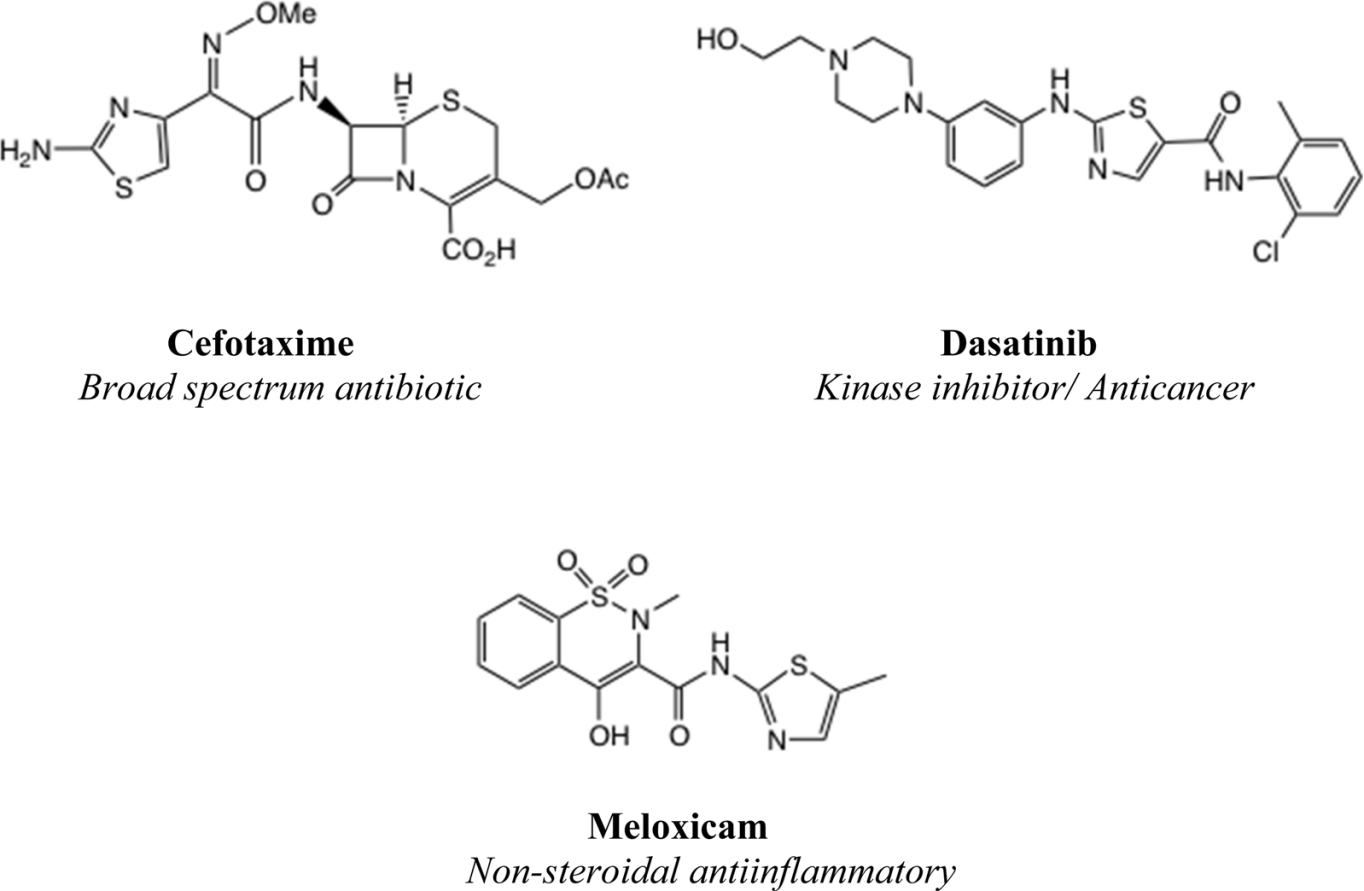
Examples of (2-aminothiazole) containing drugs.

A 2-aminothiazole unit is a common feature of many biologically active molecular series,^1^ such as cephalosporin antibiotics, kinase inhibitor anticancer agents and non-steroidal antiinflammatory drugs, Fig. 1. It has been suggested that 2-aminothiazole substitution favourably affects both the activity profile and absorption properties.^2,3^ Although a thiazole unsubstituted at both C(4) and C(5) is regarded as a metabolic risk,^4^ this danger is readily averted by appropriate substitution, especially with electron-withdrawing substituents.^5^

**Fig. 2:**
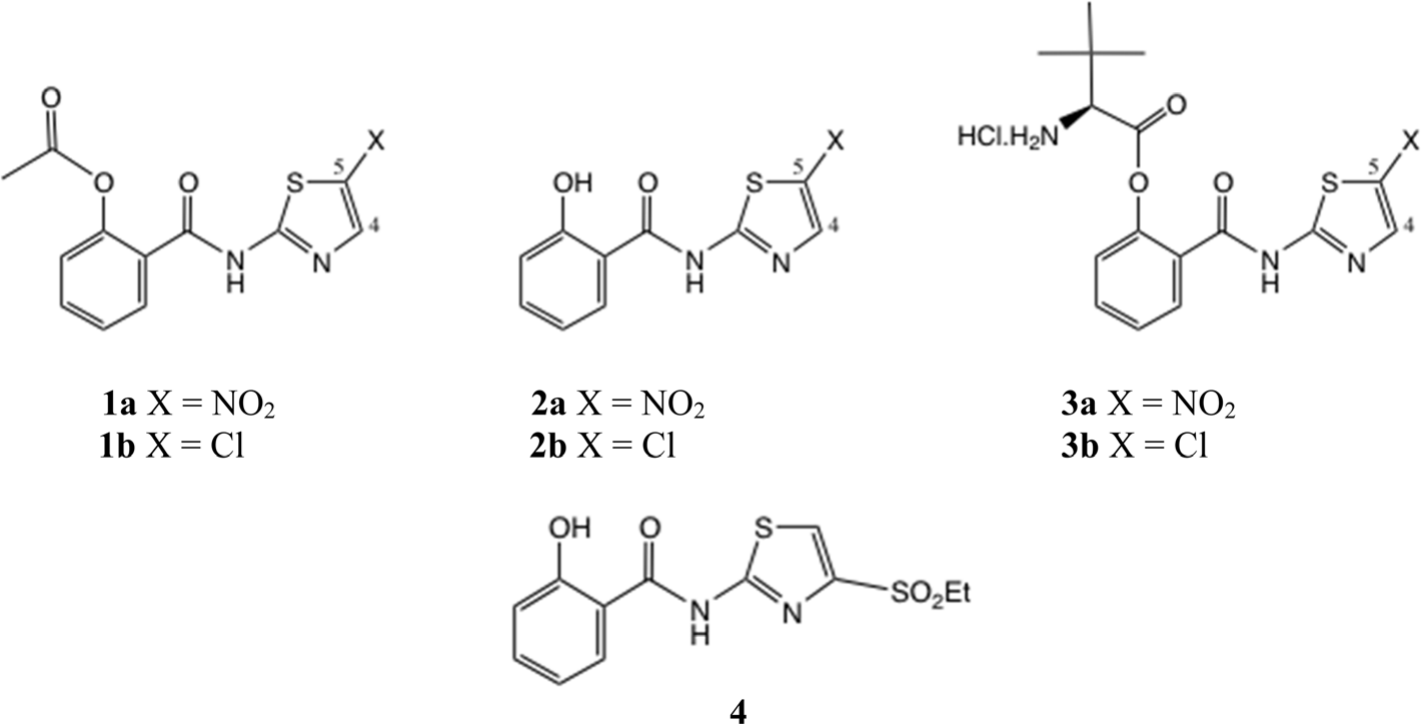
Thiazolide structures.

One important class of broad spectrum antiinfective 2-aminothiazole derivatives are the thiazolides, or [2-hydroxyaroyl-*N*-(thiazol-2-yl)-amides], typified by nitazoxanide 1a which was first reported in 1975 (Fig. 2).^6^ To this day 1a remains the antiparasitic agent of choice against *Cryptosporidium* spp.^7^ It was later discovered that 1a and other analogues, notably the 5-chloro analogue 1b, were broad-spectrum antiviral agents,^8–10^ dating from the use of 1a in treating cryptosporidiosis in AIDS patients.

We have described the structure–activity relationships (SAR) of a wide range of thiazolides against hepatitis B, hepatitis C and influenza A viruses.^11–13^ Against a typical H1N1 strain of influenza A virus, compound 1a shows IC_50_ = 3.3 μM and 1b shows IC_50_ = 3.4 μM.^13*b*^ Clinical trials of 1a have been performed against rotavirus^14^ and acute uncomplicated influenza A.^15*a*,*b*^ More recently, the SARS-CoV2 pandemic led to a strong resurgence of interest in small molecule antivirals, and NTZ has shown notable activity in trials against SARS-CoV2.^16^ The active circulating metabolites of 1a/1b*in vivo* are the free phenols 2a/2b, of which the phenolic acetates are prodrugs.^17^ Later we prepared more efficient, amino-acid ester prodrugs 3a/3b, which were shown to offer greatly improved bioavailability compared to 1a/1b.^18^

In general, 5-substituted thiazolides such as 1a/1b are the easiest to obtain. The natural position of electrophilic substitution of a 2-aminothiazole is at position 5, even when the 2-amine is acylated. In order to synthesise thiazolides with a 4-substituent, including 4-halo examples, various methods are possible: the 4-sulfonyl thiazolide 4 was synthesised from a thioester.^13*b*^

**Scheme 1:**
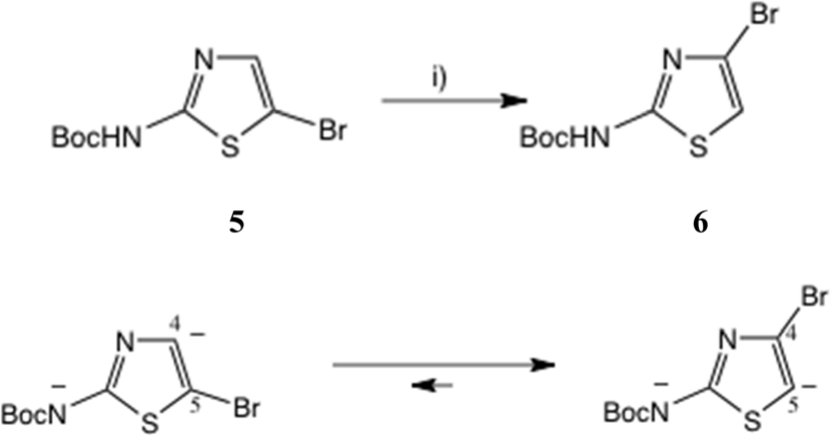
Synthesis of 2-Boc-amino, 4-bromothiazole by halogen rearrangement. Conditions: (i) LiNPr_2_^i^, THF, 0–10 °C, 20 min, 91%.

**Fig. 3:**
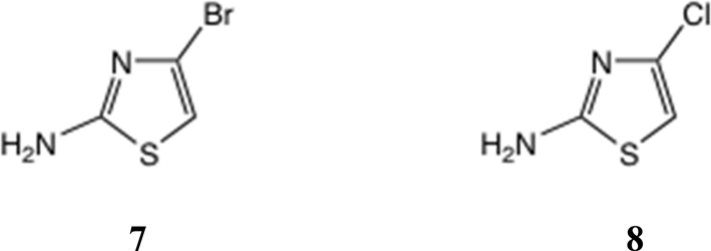
2-Amino-4-halothiazoles.

One approach to a 2-amino-4-bromothiazole uses the halogen dance rearrangement from a protected 5-Br thiazole, as originally described by Stangeland and Stanetty (Scheme 1),^19,20^ employing LiNPr_2_^i^ in THF. The rearrangement of 5 to 6 is considered to proceed *via* the *N*, *C*(5)-dianion which is thermodynamically preferred (Scheme 1, lower). This proved a robust procedure, but on removal of the Boc group the free amine 7, Fig. 3, proved rather unstable and difficult to acylate, in contrast to 2-amino-5-bromothiazole.

The literature on 2-amino-4-chlorothiazole 8 is limited,^21,22^ and here again, though we were able to reproduce one synthesis of this material in very low yield,^21^ we found 8 was unstable as the free base and difficult to acylate, giving mixed products.

The acidity of the amide NH in thiazolides such as 1a and 1b suggested an alternative route to 4′-substituted thiazolides, *viz.* further acylation of *N*-protected versions of 7 and 8, followed by mild deprotection. We now report that *t*-butyl (4-*oxo*-4,5-dihydrothiazol-2-yl)carbamate is an ideal, versatile precursor for such derivatives.

## Discussion

### Acylation of 2-amino-4-bromo and 2-amino-4-chlorothiazole

**Scheme 2:**
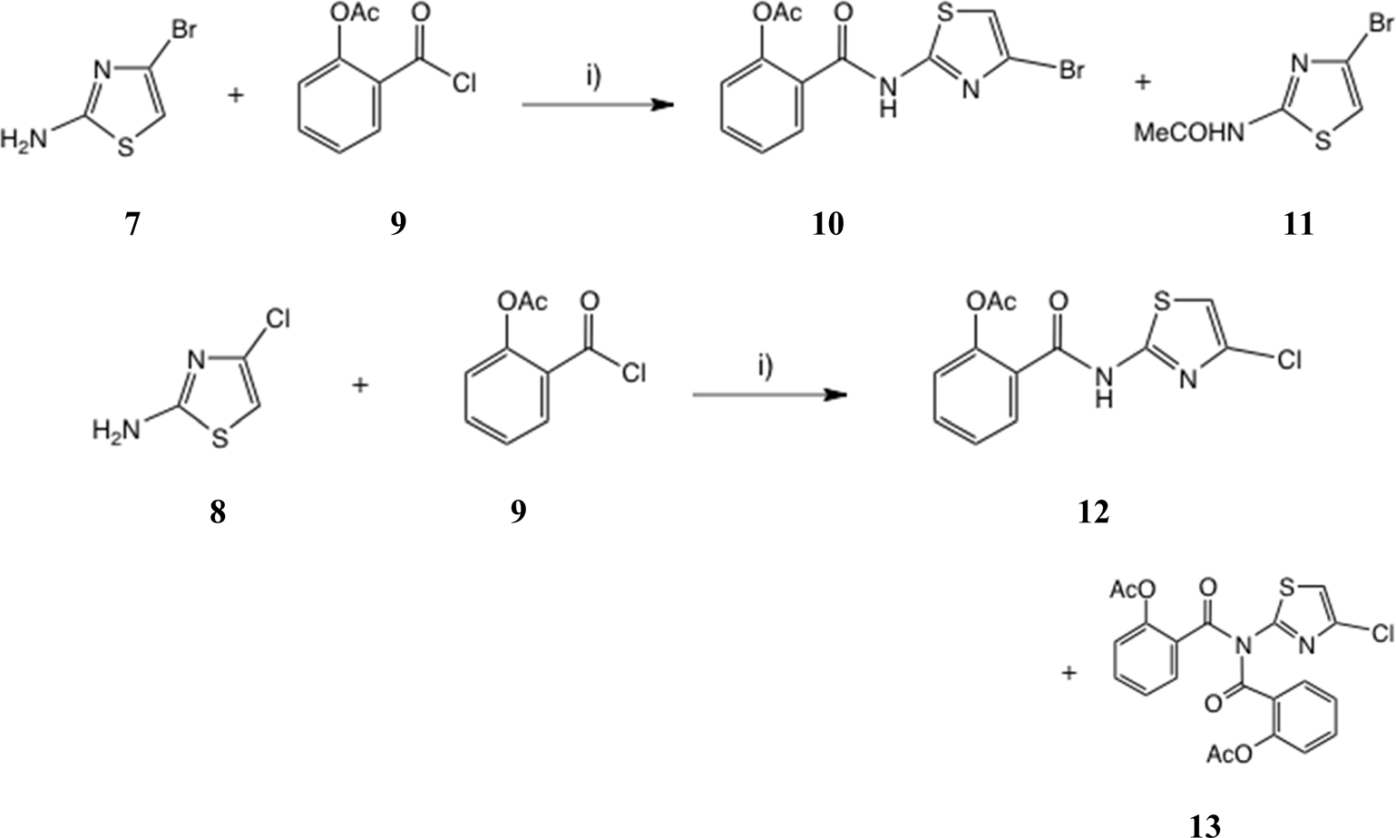
Acylation of 2-amino-4-bromo and 2-amino-4-chlorothiazole. Conditions: (i) THF, Et_3_N, 0 °C–20 °C.

Treatment of Boc derivative 6 ^20^ with TFA in CH_2_Cl_2_, followed by basification with NaHCO_3_ and extraction, afforded the free amine 7 in 94% yield, which proved rather unstable on storage and was used immediately, Scheme 2. Reaction of 7 with *O*-acetylsalicyloyl chloride 9 using two-phase acylation conditions^11,12^ was quite unsuccessful. Instead, anhydrous acylation in THF using Et_3_N as base gave a slow, complex reaction. Workup after 46 h at 20 °C gave two major products by chromatography, from which the desired thiazolide 10 was isolated in 17% yield and purified by recrystallisation. A major byproduct was apparently the acetamide 11 (*m*/*z* 221, 223) though this was difficult to purify fully.

**Fig. 4:**
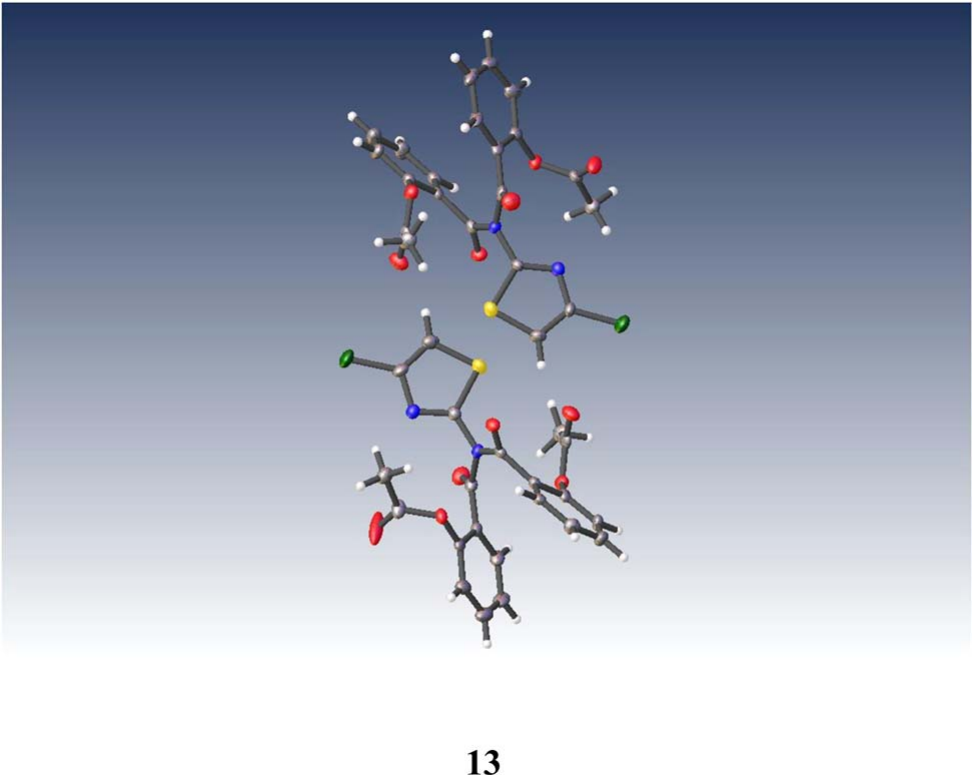
Single crystal X-ray structure of (((4-chlorothiazol-2-yl)azanediyl)bis(carbonyl))bis(2,1-phenylene) diacetate 13.

Similarly, anhydrous acylation of 2-amino-4-chlorothiazole 8 ^21^ with 9 again gave a slow complex reaction. By chromatography, the desired thiazolide 12 was obtained in 19% yield and further purified by recrystallisation. A significant more polar product proved to be a bis-acylated derivative 13, which interestingly possessed a bis-acylamino rather than a tautomeric acylimino structure, as shown by single crystal X-ray analysis, Fig. 4. We therefore turned to alternative 2-aminothiazole intermediates.

### Protected forms of pseudothiohydantoin

#### *N*-(4-*Oxo*-4,5-dihydrothiazol-2-yl)acetamide and its chlorination

Pseudothiohydantoin 14, sc. 2-aminothiazol-4(5*H*)-one, is commercially available or easily prepared from thiourea and bromoacetic acid in a typical Hantzsch synthesis,^23^ and carries built-in 4-substitution. As noted above, heating 14 with excess POCl_3_ ^21^ gave a very low yield of 2-amino-4-chlorothiazole 8.

**Scheme 3:**
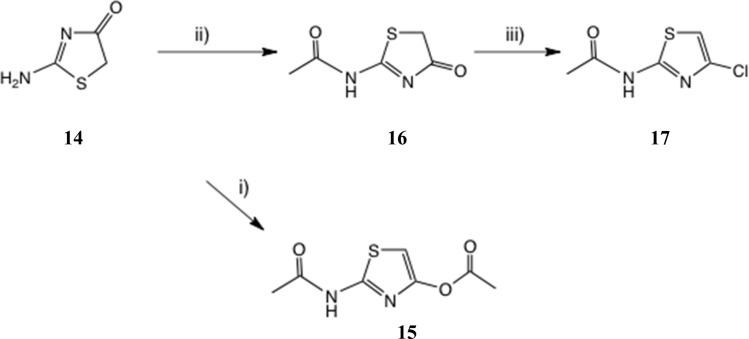
(2-Acetamido)thiazole intermediates. Conditions: (i) Ac_2_O, DMAP, THF, 0–20 °C, 22 h, 81%; (ii) Ac_2_O, *N*–Me morpholine, THF, 60 °C, 1.5 h, 83%; (iii) POCl_3_, MeCN, 50 °C, 3 h, 57% or NCS, Ph_3_P, MeCN, 20 °C, 5 h, 72%.

**Fig. 5:**
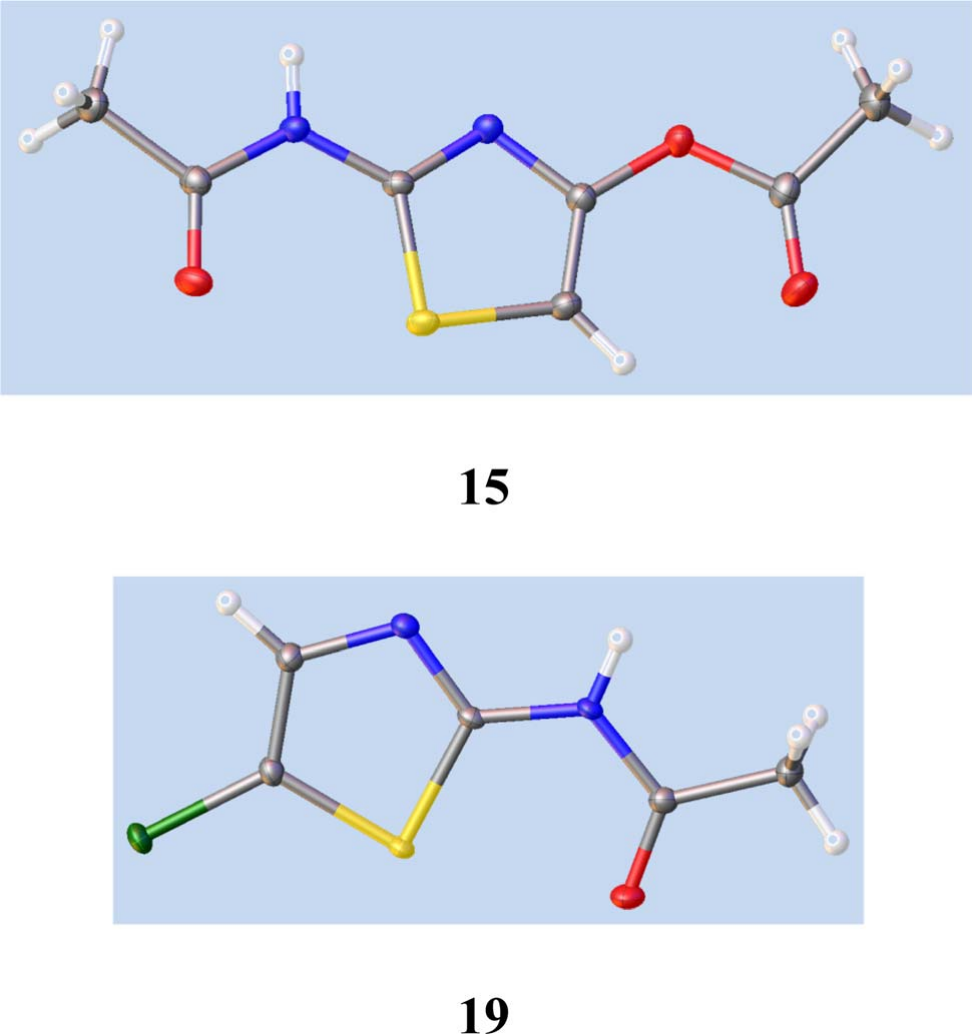
Single crystal X-ray structures of 15 and 19. See ESI† for cif file data. The *syn*-orientation of the S atom and carbonyl oxygen in both cases results from nonbonding overlap between the C–S σ* orbital and O lone pair electrons.^26^

We therefore studied *N*-protected versions of 14, aiming first at the acetamide, Scheme 3. Heating 14 with Ac_2_O/AcOH^24^ led to a very slow reaction, even at 100–105 °C, so we switched to amine bases. Treatment of 14 with Ac_2_O and DMAP in THF at 20 °C gave a steady reaction and delivered very largely the previously unknown *N*, *O*-diacetate 15 in high yield; its structure was confirmed by a single crystal X-ray determination, Fig. 5, since other tautomeric products were possible. The use of Et_3_N gave a mixture of products including 15 and the desired monoacetamide.

Use of the weaker base *N*-methylmorpholine at 60 °C gave a controlled reaction, which generated the desired acetamide 16 in very good yield with negligible diacetylation. Treatment of 16 with POCl_3_ at 50 °C gave a very slow reaction until catalytic DMF was added; the 4-Cl compound 17 ^25*a*^ was then isolated in satisfactory yield. The same product was obtained in 72% yield by reaction of 16 with Ph_3_P and *N*-chlorosuccinimide (NCS) (1.5 eq. each; *cf.* next section) in MeCN at 20 °C. Another route claims chlorination of 2-acetamidothiazole using ‘green’ conditions, *viz.* NaCl and oxone,^25*b*^ but it is not clear whether the 4-Cl isomer 17 is the product since these authors' NMR data look significantly different from ours. The reaction of 2-aminothiazole with 1-chloro-1,2-benziodoxol-3-one^22^ was also stated to afford 17.

**Scheme 4:**
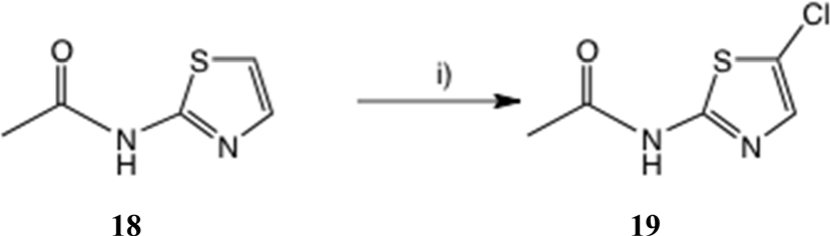
Synthesis of 2-acetamido-5-chlorothiazole. Conditions: (i) NCS, Amberlite A-15 (H^+^), 20 °C, 22 h, 65%.

To seek reassurance on the regiochemical point, we studied the direct chlorination of 2-acetamidothiazole 18 with NCS in MeCN, Scheme 4. We used a similar procedure once before on a thiazolide.^11^ In fact this chlorination proceeded smoothly, using mild acid catalysis with Amberlyst A-15 (H^+^) resin, and the product, isolated in unoptimised 65% yield, was shown to be the 5-Cl isomer 19 by a single crystal X-ray determination, Fig. 5. The ^1^H and ^13^C NMR data of this material were identical with those reported^25*b*^ and claimed to be the 4-Cl isomer.

Under relatively mild conditions (HCl, aq. MeOH, 50 °C) we found that hydrolysis of 17 gave rapid decomposition with reversion to 14. This probably resulted from ring protonation at C(5) followed by attack of water at C(4).

#### *Tert*-Butyl (4-*oxo*-4,5-dihydrothiazol-2-yl)carbamate and its halogenation

**Scheme 5:**
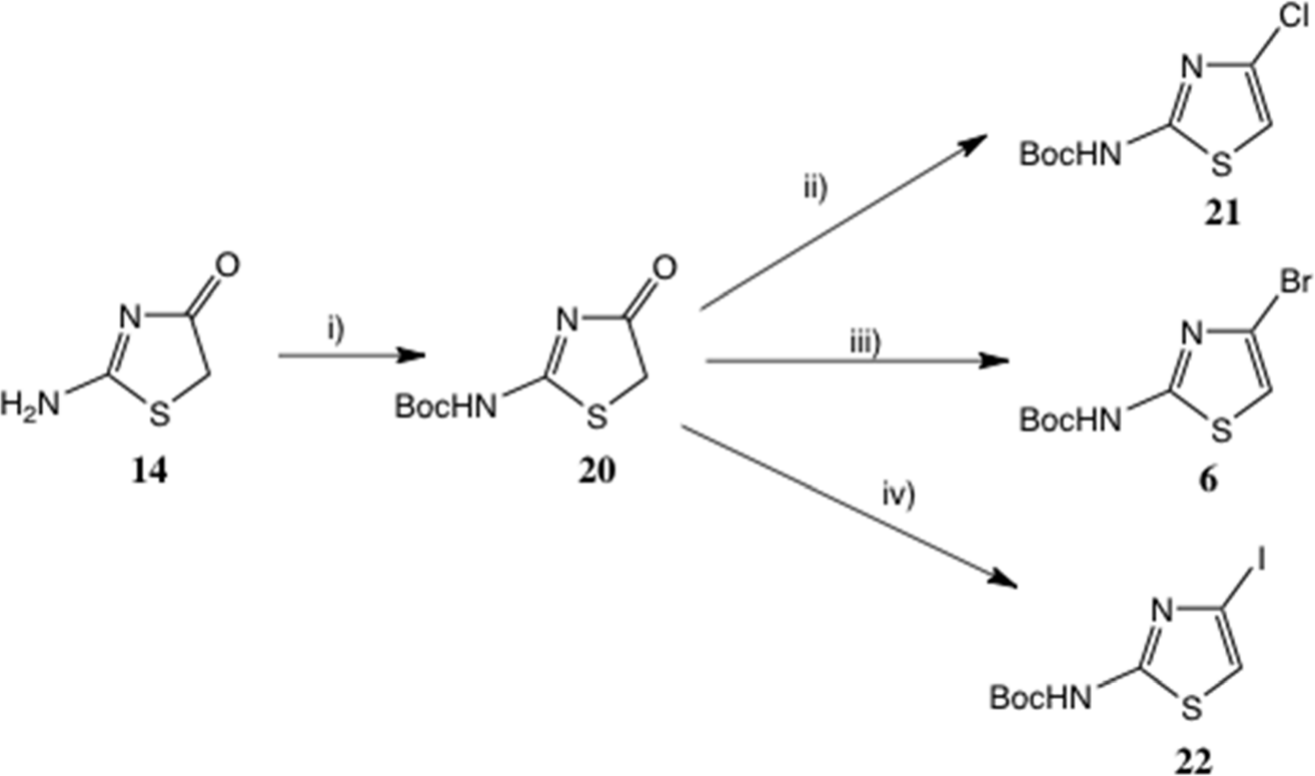
Conversion of pseudothiohydantoin to 2-Boc-amino-4-halothiazoles. Conditions: (i) Boc_2_O, aq. THF, pH10, 86%; (ii) Ph_3_P, Cl_3_CCN, CH_2_Cl_2_, 73%; (iii) Ph_3_P, NBS, MeCN, 63%; (iv) Ph_3_P, NIS, MeCN, 28%. Alternative conditions discussed in text.

We therefore switched to Boc protection, to allow for mild *anhydrous* acidolysis eventually. Boc pseudothiohydantoin is disclosed in the patent literature,^27*a*^ prepared by reaction of di-*t*-butyl pyrocarbonate (Boc_2_O) with 14 in 15% yield using DMAP catalysis. Instead, using THF-water at pH 10 with Na_2_CO_3_ or NaOH, a clean conversion to the mono-Boc derivative was obtained: 20 was isolated in 86% yield, Scheme 5. Under these conditions, formation of any bis-adduct is minimal and excess Boc_2_O is steadily hydrolysed. A later Pfizer patent^27*b*^ cited a similar yield by heating 14 with two equivalents of Boc_2_O and no catalyst in THF at 60 °C for 48 h.

We anticipated that 20 would be readily enolised; hence reagents for the chlorination of other tautomeric hydroxy heterocycles such as 2-hydroxypyridine/2-pyridone under Appel-type conditions^28^ should be effective. More recently, variants of the original Appel method using catalytic Ph_3_PO^29^ and a sustainable procedure avoiding chlorinated solvents^30^ have been described. Here, Ph_3_P in conjunction with CCl_4_ ^31^ (or preformed Ph_3_PCl_2_ ^32^), *N*-chlorosuccinimide^33^ or trichloroacetonitrile^34^ all converted 20 into 21. THF, CH_2_Cl_2_ and MeCN were all adequate solvents; the best and mildest conditions proved to be Ph_3_P and Cl_3_C·CN in CH_2_Cl_2_ at 20 °C, affording 21 in 73% yield.

For the introduction of Br at C(4), as noted earlier, the Br rearrangement (‘halogen dance’)^20*a*,*b*^ is feasible: the substrate (Scheme 1) is prepared from 2-amino-5-bromothiazole.^35^ Here too, however, 20 proved a highly suitable intermediate, and on treatment with Ph_3_P and *N*-bromosuccinimide^33,34*b*^6 was readily obtained.^36^ Here the solvent choice was significant, with MeCN definitely superior to CH_2_Cl_2_, giving 6 in 63% yield. Another good reagent proved to be ethyl tribromoacetate,^34*b*,37^ again employing MeCN, which gave a virtually identical yield, though here purification was more difficult. It is noteworthy that MeCN often proves a superior solvent in the Appel-type halogenation reaction^38^ and may even divert the reaction to other products.^39^

2-Boc-amino-4-iodothiazole 22 was disclosed in a patent^40^ as a useful intermediate for Suzuki couplings, but with no preparative detail. We obtained this compound in an unoptimised 28% yield by treatment of 20 with Ph_3_P and *N*-iodosuccinimide at 0–20 °C; a little free I_2_ was used to initiate the reaction.^41^

**Scheme 6:**
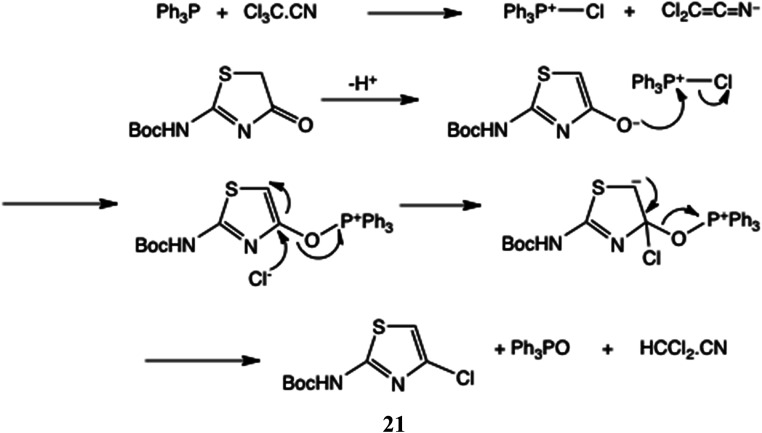
Proposed halogenation mechanism with Cl_3_C·CN.

In Scheme 6 we give a mechanism for these halogenations, using Cl_3_C·CN as the example donor, generating 21.

**Scheme 7:**
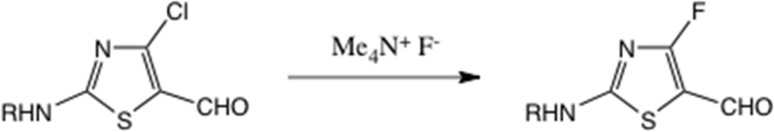
Synthesis of a 4-fluorothiazole by nucleophilic halogen substitution; R=Ac or Boc.

We also studied the reactions of both 16 and 20 with Middleton's DAST reagent,^42^ hoping to gain access to 4-fluoro derivatives: currently there is no reported preparation of 2-amino-4-fluorothiazole. Neither gave useful products; the reaction of 16 gave a low yield of a complex mixture and 20 gave rapid loss of the Boc group. A 4-fluorothiazole bearing a 5-formyl substituent was recently obtained^23^ by displacement from a 4-chlorothiazole using S_N_Ar reaction with anhydrous Me_4_N^+^ F^−^, Scheme 7.

### Acylation of Boc intermediates and thiazolide synthesis

**Scheme 8:**
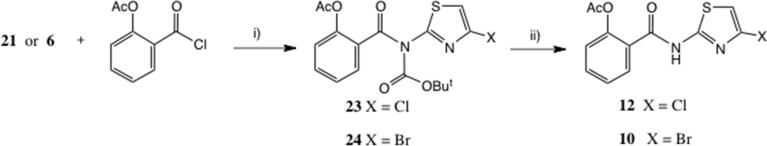
Thiazolide synthesis. Conditions: (i) Et_3_N, THF, 61% for 23, 70% for 24; (ii) dil. CF_3_CO_2_H, CH_2_Cl_2_, 97% for 12, 65% for 10.

The NH of compounds such as 21 is considerably more acidic than a typical amide^43^ or even acetanilide (p*K*_a_ = 13),^44^ and our previous experience had indeed shown that further *N*-acylation was possible. Using Et_3_N as base, acylation of 21 with *O*-acetylsalicyloyl chloride cleanly afforded a 70% yield of the Boc intermediate 23 (Scheme 8). Mild acidolysis (dilute CF_3_CO_2_H, CH_2_Cl_2_) then delivered thiazolide 12 in near quantitative yield, identical to the product obtained in low yield by acylation of 8, Scheme 2.

This sequence was equally applicable to the bromo intermediate 6, which *via* intermediate 24 gave 10, *cf.*Scheme 2. Clearly this sequence represents the method of choice for the synthesis of 10 and 12.

## Conclusions

*N*-Boc protected forms of 2-amino-4-halothiazoles are readily available from Boc-pseudothiohydantoin, which is itself available from pseudothiohydantoin in high yield. The tendency of the heterocycle to exhibit tautomeric behaviour and to overreact with electrophiles is thus avoided. In general, *N*-halosuccinimides in conjunction with Ph_3_P under Appel-type conditions are effective reagents for the halogenation step, but Cl_3_CCN proved optimal for chlorination. Further acylation of these intermediates with *O*-acetylsalicyloyl chloride, followed by mild deprotection, offers high-yielding syntheses of 4-bromo and 4-chlorothiazolides. The relatively high acidity of amide NHs in derivatives such as 21 is significant: this bis-acylation/mild deprotection sequence may well offer good alternative syntheses for other heterocyclic amides. Direct acylation of the corresponding free amines 7 and 8, by contrast, gave low yields of mixed products.

## Experimental

### General experimental procedures

Organic extracts were finally washed with saturated brine and dried over anhydrous Na_2_SO_4_ prior to rotary evaporation at <30 °C. Moisture sensitive reactions were carried out in anhydrous organic solvents (purchased from Sigma-Aldrich) under a N_2_ or Ar atmosphere. Reactions were monitored by analytical thin-layer chromatography using Merck Kieselgel 60 F_254_ silica plates, and were viewed under UV or by staining with KMnO_4_ or iodine. Preparative flash column chromatography was performed on either VWR Prolabo silica gel or Sigma-Aldrich silica gel (particle size 40–63 Å). Melting points were recorded using a Bibby-Sterlin Stuart SMP3 melting point apparatus and are uncorrected. Mass spectra were obtained in either electrospray mode (ES) with a Micromass LCT or chemical ionization (CI) mode with a Micromass Trio 1000 using ammonia. Elemental analyses were performed by Mrs Jean Ellis, University of Liverpool. ^1^H and ^13^C NMR spectra were obtained using a Bruker Avance or a Bruker DPX 400 instrument operating at 400 and 100 MHz, respectively; chemical shifts are reported in ppm (*δ*) relative to Me_4_Si. Coupling constants (*J*) are reported in Hz.

### 2-Amino-4-bromothiazole 7

A solution of *tert*-butyl (4-bromothiazol-2-yl)carbamate 6 ^20^ (0.56 g, 2 mmol) in CH_2_Cl_2_ (8 mL) was stirred at 20 °C with CF_3_CO_2_H (5 mL). After 4 h, the solution was evaporated to dryness, azeotroped with CH_2_Cl_2_ (2 × 5 mL) and the residue was partitioned between satd. aq. NaHCO_3_ (20 mL) and CH_2_Cl_2_ (5 × 10 mL). Evaporation gave 7 as a white solid (0.336 g, 94%) which was progressed immediately; *δ*_H_ (CDCl_3_) 5.32 (2H, br s, NH_2_) and 6.41 (1H, s, 5-H).

### 2-((4-Bromothiazol-2-yl)carbamoyl)phenyl acetate 10

Method A: a solution of 2-amino-4-bromothiazole 7 (0.42 g, 2.35 mmol) and *O*-acetylsalicyloyl chloride 9 (0.93 g, 4.69 mmol) in dry THF (10 mL) was stirred under N_2_ at 0 °C and Et_3_N (0.82 mL, 5.88 mmol) was added. The mixture was allowed to regain 20 °C, then after 23 h, 4- *N*,*N*-dimethylaminopyridine (0.12 g, 1 mmol) was added. After a total of 46 h, the mixture was diluted with EtOAc (20 mL) and worked up for a neutral product (0.93 g), which was chromatographed, eluting with a gradient of 30–50% EtOAc-*n*-hexane. Evaporation of early-eluting fractions afforded title compound 10 (0.133 g) which was recrystallised from EtOAc-*n*-hexane to afford pure product (0.100 g, 12.5%), m.p. 148–150 °C. Found: C, 42.3; H, 2.97; N, 7.96; S, 9.39; *m*/*z*, 362.9416. C_12_H_9_BrN_2_O_3_S requires C, 42.25; H, 2.7; N, 8.2; S, 9.4%; *m*/*z*, 362.9415 (MNa^+^); *δ*_H_ (CDCl_3_) 2.46 (3H, s, CH_3_CO), 6.92 (1H, s, thiazole 5-H), 7.40 (1H, d, Ar H), 7.63 (2H, m, Ar H), 8.04 (1H, d, Ar H) and 9.94 (1H, s, NH); *δ*_C_ (CDCl_3_) 21.3, 111.8, 121.3, 123.8, 124.4, 126.8, 130.9, 133.8, 148.5, 158.2, 162.4 and 168.3.

Later column fractions were pooled and evaporated to give a white solid (0.332 g) whose spectroscopic data were consistent with the amide 11 plus other traces; *δ*_H_ (CDCl_3_) 2.33 (3H, s, CH_3_CO), 6.88 (1H, s, 5-H) and 10.65 (1H, br s, NH); found: *m*/*z* (CI, methane) 220.9387; C_5_H_6_^79^BrN_2_OS (MH^+^) requires *m*/*z*, 220.9379.

Method B: a solution of Boc derivative 24 (0.292 g, 0.64 mmol, v. i.) in CH_2_Cl_2_ (2 mL) was stirred at 20 °C and CF_3_CO_2_H (0.3 mL, 4 mmol) was added dropwise. After 3 h, the solution was diluted with EtOAc (20 mL) and cautiously washed with satd. NaHCO_3_ (10 mL). Standard workup afforded the title compound 10 as a white solid, essentially pure (0.147 g, 65%), m.p. 148–150 °C. Analytical and spectroscopic data were identical to those obtained by Method A.

### 2-((4-Chlorothiazol-2-yl)carbamoyl)phenyl acetate 12 and {[(4-chlorothiazol-2-yl)azanediyl]bis(carbonyl)]bis(2,1-phenylene} diacetate 13

Method A: 2-amino-4-chlorothiazole 8 ^21^ (0.135 g, 1 mmol) was dissolved in anhydrous THF (4 mL) and stirred at 20 °C with *O*-acetylsalicyloyl chloride 9 (0.24 g, 1.2 mmol) under N_2_. After addition of triethylamine (0.21 mL, 1.5 mmol), stirring was continued for 28 h then further acid chloride and triethylamine (1 mmol each) were added. After 96 h in all, the reaction was diluted with EtOAc (30 mL) and worked up for a neutral product, giving a pale orange gum (0.424 g). Chromatography, eluting with a gradient of 25–33% EtOAc in hexane, afforded firstly the mono-amide 12 (0.074 g, 25%), mp 148–149 °C. Found: C, 48.6; H, 3.0; N, 9.4; S, 10.6; *m*/*z* (ES +ve mode) 318.9918; C_12_H_9_ClN_2_O_3_S requires C, 48.6; H, 3.1; N, 9.4; S, 10.8%; C_12_H_9_^35^ClN_2_O_3_SNa (MNa^+^) requires *m*/*z*, 318.9920; *δ*_H_ (CDCl_3_) 2.48 (3H, s, CH_3_CO), 6.79 (1H, s, thiazole 5-H), 7.26 (1H, d, ArH), 7.42 (1H, t, ArH), 7.62 (1H, t, ArH), 8.07 (1H, d, ArH) and 9.92 (1H, br s, NH); *δ*_C_ (CDCl_3_) 21.3, 108.1, 123.8, 124.3, 126.8, 131.0, 133.9, 135.4, 148.4, 157.2, 162.3 and 168.2. Later column fractions were pooled and evaporated to afford the bis-amide 13 (0.077 g, 17%), m. p. 111–112 °C. Found: C, 55.0; H, 3.3; N, 6.1; S, 6.7; *m*/*z* (ES +ve mode) 481.0230; C_21_H_15_ClN_2_O_6_S requires C, 55.0; H, 3.3; N, 6.1; S, 7.0%; C_21_H_15_^35^ClN_2_O_6_SNa (MNa^+^) requires *m*/*z*, 481.0237; *δ*_H_ (CDCl_3_) 2.35 (3H, s, CH_3_CO), 7.07 (1H, s, thiazole 5-H), 7.08 (1H, d, ArH), 7.16 (1H, t, ArH), 7.42 (1H, t, ArH) and 7.57 (1H, d, ArH); *δ*_C_ (CDCl_3_) 21.1 (×2), 113.7 (×2), 123.4 (×2), 125.9, 126.8, 130.3, 133.5, 136.9, 148.5, 157.7, 167.2 and 168.7. Recrystallisation of 13 gave material of excellent crystalline form suitable for single crystal X-ray determination, q. v.

Method B: the *N*-Boc intermediate 23 (0.171 g, 0.43 mmol, v. i.) was dissolved in CH_2_Cl_2_ (3 mL) and stirred at 20 °C, then CF_3_CO_2_H (0.5 mL) was added over 1 min. Complete reaction was observed after 1 h; the solution was diluted with EtOAc (20 mL) and washed with satd. aq. NaHCO_3_ (20 mL), giving an aq. pH∼8, then the organic phase was further washed with water and evaporated to give the product 12 (0.124 g, 97%) as a white solid. Analytical and spectroscopic data were identical to those obtained by Method A.

### *N*-(4-*Oxo*-4,5-dihydrothiazol-2-yl)acetamide 15

A suspension of pseudothiohydantoin 14 (0.50 g, 4.31 mmol) in THF (4 mL) and Ac_2_O (1 mL) was stirred at 20 °C during addition of *N*–Me morpholine (1 mL), then heated at 65 °C for 1.5 h, when much solid had deposited. The mixture was cooled, treated with Et_2_O (10 mL) and stored at 0 °C for 1 h, then filtered, washed with Et_2_O, dried and evaporated to give essentially pure product 15 as a pale brown solid (0.565 g, 83%); an analytical sample was obtained by recrystallisation from MeOH-EtOAc. Found: C, 38.1; H, 3.8; N, 17.8; S, 20.15; *m*/*z* (CI, CH_4_) 159.0228. C_5_H_6_N_2_O_2_S requires C, 38.0; H, 3.8; N, 17.7; S, 20.3%; C_5_H_7_N_2_O_2_S (MH^+^) requires *m*/*z*, 159.0223; *δ*_H_ (d_6_-DMSO) *δ* 2.19 (3H, s, CH_3_CO), 3.85 (2H, s, CH_2_CO) and 12.61 (1H, br s, NH); *δ*_C_ (d_6_-DMSO) 24.3, 37.1, 173.0, 182.6 and 188.2.

### 2-Acetamidothiazol-4-yl acetate 16

A suspension of pseudothiohydantoin 14 (0.5 g, 4.31 mmol) in THF (4 mL) and Ac_2_O (1 mL) was stirred at 20 °C and *N*,*N*-dimethylaminopyridine (0.61 g, 5 mmol) was added. A yellow-orange solution gradually resulted, and after 6 h the mixture was stored at 0 °C for 16 h, then partitioned between EtOAc (30 mL + 10 mL) and 7% aq. citric acid (25 mL). The combined extracts were washed with brine, dried and evaporated to give the title compound 16 as a near-white solid (0.70 g, 81%). Found: C, 42.3; H, 4.0; N, 13.6; S, 15.8; *m*/*z* (CI, CH_4_) 201.033; C_7_H_8_N_2_O_3_S requires C, 42.0; H, 4.0; N, 14.0; S, 16.0%; C_7_H_9_N_2_O_3_S (MH^+^) requires *m*/*z*, 201.0328; *δ*_H_ (d_6_-DMSO) *δ* 2.14, 2.26 (6H, 2s, 2xCH_3_CO), 6.73 (1H, 5-H) and 12.18 (1H, br s, NH); *δ*_C_ (d_6_-DMSO) 21.1, 23.0, 97.1, 149.9, 156.2, 168.7 and 169.5.

### *N*-(4-Chlorothiazol-2-yl)acetamide 17

A mixture of the acetamide 15 (0.40 g, 2.5 mmol) and POCl_3_ (1 mL) in MeCN (4 mL) was heated with stirring at 50 °C; reaction was initiated by addition of DMF (3 drops). After 3 h, the mixture was cooled and partitioned between EtOAc (30 mL + 10 mL) and 10% aq. Na_2_CO_3_ which was added cautiously to give a pH of 8. The combined organic extracts were washed with H_2_O, brine, dried and evaporated to give the title compound 17 (0.256 g, 57%) as a pale yellow solid, m. p. 144–145 °C (from EtOAc-hexane). Found: *m*/*z* (CI, CH_4_) 176.9892. C_5_H_6_^35^ClN_2_OS (MH^+^) requires *m*/*z*, 176.9884; *δ*_H_ (d_6_-DMSO) *δ* 2.15 (3H, s, CH_3_CO), 7.14 (1H, s, 5-H) and 12.36 (1H, br s, NH); (CDCl_3_) 2.35 (3H, s, CH_3_CO), 6.77 (1H, s, 5-H) and 10.93 (1H, br s, NH); *δ*_C_ (d_6_-DMSO) 22.9, 108.0, 133.8, 158.7 and 169.5; (CDCl_3_) 23.4, 107.7, 134.1, 159.3 and 168.7.

### *N*-(5-Chlorothiazol-2-yl)acetamide 19

A solution of 2-acetamidothiazole 18 (0.28 g, 2 mmol) in acetonitrile (5 mL) was stirred with *N*-chlorosuccimimide (0.27 g, 2.00 mmol) over Amberlite A-15 (H^+^) (0.5 g) at 20 °C. After 22 h, when much white solid had been deposited, EtOAc (40 mL) was added to give a clear solution which was decanted from the resin, washed with water and brine, dried over Na_2_SO_4_ and evaporated to give 19 as a white solid (0.229 g, 65%). Recrystallisation from EtOAc-hexane afforded material suitable for a single crystal X-ray structure determination, see Fig. 1, m. p. 200–202 °C (softened ∼190 °C). Found: C, 33.9; H, 2.8; N, 15.8. C_5_H_5_ClN_2_OS requires C, 34.00; H, 2.85; N, 15.86%; *δ*_H_ 2.33 (3H, s, CH_3_CO), 7.27 (1H, s, 4-H) and 11.80 (1H, br s, NH); *δ*_C_ 22.9, 121.0, 133.5, 157.6 and 168.0.

### *Tert*-Butyl (4-*oxo*-4,5-dihydrothiazol-2-yl)carbamate 20

To a solution of pseudothiohydantoin 14 (1.0 g, 8.6 mmol) in 1 : 1 H_2_O : THF (15 mL) was added Boc_2_O (2.4 g, 11 mmol) followed by portionwise addition of NaOH (0.88 g, 22 mmol). The reaction mixture was stirred at 20 °C for 16 h, then partitioned between 7% aq. citric acid (30 mL) and CH_2_Cl_2_ (3 × 25 mL). The combined organic extracts were dried over MgSO_4_, filtered and evaporated to dryness, to afford the title product 20 (1.60 g, 86%) as a pale-yellow solid which was sufficiently pure to progress directly; an analytical sample was obtained by recrystallisation from EtOAc-hexane, 1 : 1. Found: C, 44.5; H, 5.6; N, 12.9; S, 14.9; *m*/*z* (ES +ve mode), 239.0461. C_8_H_12_N_2_O_5_S requires C, 44.4; H, 5.6; N, 12.95; S, 14.8%; C_8_H_12_N_2_O_5_SNa (MNa^+^) requires *m*/*z*, 239.0461; *δ*_H_ (CDCl_3_) *δ* 1.55 (s, 9H), 3.80 (s, 2H) and 9.61 (br s, 1H, NH); *δ*_C_ (CDCl_3_) *δ* 27.9, 36.4, 84.4, 153.6, 181.8 and 183.3.

### *tert*-Butyl (4-chlorothiazol-2-yl)carbamate 21

A solution of carbamate 20 (0.54 g, 2.50 mmol) and triphenylphosphine (0.98 g, 3.75 mmol) in anhydrous CH_2_Cl_2_ (7.5 mL) was stirred at 20 °C under N_2_ and trichloroacetonitrile (0.38 mL, 3.75 mmol) was added over one minute. After 40 h the mixture was diluted with Et_2_O (30 mL), washed with H_2_O (2×), brine, dried and evaporated to a clear gum. Chromatography, eluting with 20%EtOAc-hexane, afforded on evaporation of appropriate fractions the title compound 21 (0.425 g, 73%) as a white solid. Found: C, 41.0; H, 4.7; N, 11.9; S, 13.7; *m*/*z* (ES +ve mode), 257.0126. C_8_H_11_ClN_2_O_2_S requires C, 40.9; H, 4.7; N, 11.9; S, 13.7%; C_8_H_11_^35^ClN_2_O_2_SNa (MNa^+^) requires *m*/*z*, 257.0122; *δ*_H_ (CDCl_3_) 1.54 (9H, s, Me_3_C), 6.65 (1H, s, 5-H) and 9.20 (1H, br s, NH); *δ*_C_ (CDCl_3_) 28.2, 83.0, 106.4, 134.4, 152.5 and 160.9.

### *tert*-Butyl (4-bromothiazol-2-yl)carbamate 6 (ref. 20)

A solution of *N*-bromosuccinimide (0.32 g, 1.77 mmol, 1.5 eq.) in anhydrous MeCN (2.0 mL) was added dropwise to a suspension of the carbamate 20 (0.25 g, 1.17 mmol) and triphenylphosphine (0.46 g, 1.74 mmol) in the same solvent (3.0 mL) with stirring under N_2_, then the reaction was stirred at 20 °C overnight. The reaction was quenched with H_2_O (20 mL) and extracted with ethyl acetate (3 × 25 mL). The combined organic extracts were washed with brine and dried over MgSO_4_. Evaporation followed by column chromatography (5–10% ethyl acetate/hexane) afforded the title compound 6 as an off-white solid (0.20 g, 63% yield). Found: (ES +ve mode) *m*/*z*, 300.9618. C_8_H_11_^79^BrN_2_NaO_2_S (MNa^+^) requires *m*/*z*, 300.9617; 1H NMR *δ*_H_ (CDCl_3_) 1.54 (9H, s, Me_3_C), 6.79 (1H, s, 5-H) and 9.50 (1H, br s, NH); *δ*_C_ (CDCl_3_) 28.2, 83.2, 110.3, 120.4, 152.2 and 161.17.

### *tert*-Butyl (4-iodothiazol-2-yl)carbamate 22

A solution of the carbamate 20 (0.22 g, 1 mmol) and Ph_3_P (0.39 g, 1.5 mmol) in MeCN (5 mL) was treated with *N*-iodosuccinimide (0.34 g, 1.5 mmol) at 0 °C and allowed to warm to 20 °C. No reaction occurred until I_2_ (0.25 g, 1 mmol) and further Ph_3_P (0.26 g, 1 mmol) were added with continued stirring at 20 °C. After a total of 22 h, EtOAc (30 mL) was added and the solution was washed with 5% aq. Na_2_S_2_O_3_ (20 mL), water and brine, then dried and evaporated to a near colourless residue. Chromatography using 20% EtOAc-hexane afforded on evaporation of appropriate fractions the iodo compound 22 as a white crystalline solid (0.091 g, 28%). Recrystallisation from EtOAc-hexane afforded an analytical sample. Found: C, 29.7; H, 3.4; N, 8.8; S, 9.4. *m*/*z* (ES +ve mode): 348.9479. C_8_H_11_N_2_O_2_SI requires C, 29.5; H, 3.4; N, 8.6; S, 9.8%; C_8_H_11_IN_2_O_2_SNa (MNa^+^) requires *m*/*z*, 348.9478; *δ*_H_ (CDCl_3_). 1.55 (9H, s, Me_3_C), 7.01 (1H, s, 5-H) and 8.8–9.2 (1H, br, NH); *δ*_C_ (CDCl_3_) 28.2, 83.3, 88.8, 117.6, 151.9 and 161.4.

### 2-[(*tert*-Butoxycarbonyl)(4-chlorothiazol-2-yl)carbamoyl]phenyl acetate 23

Compound 21 (0.175 g, 0.75 mmol) and *O*-acetylsalicyloyl chloride 9 (0.15 g, 0.75 mmol) were stirred in anhydrous THF (3 mL) under N_2_ at 20 °C, then triethylamine (0.14 mL, 1 mmol) was added. After 22 h, when most 21 had reacted and some solid had been deposited, the mixture was diluted with EtOAc (20 mL) then washed successively with 7% aq. citric acid, satd. aq. NaHCO_3_ and water, then evaporated to give a sticky solid (0.295 g). Chromatography, eluting with 20% EtOAc-hexane, afforded on evaporation of appropriate fractions the title compound 23 (0.181 g, 61%) as a white solid. Found: C, 51.3; H, 4.2; N, 6.9; S, 7.4; *m*/*z* (ES +ve mode) 419.0444; C_17_H_17_ClN_2_O_5_S requires C, 51.45; H, 4.3; N, 7.1; S, 8.1%; C_17_H_17_^35^ClN_2_O_5_SNa (MNa^+^) requires *m*/*z*, 419.0439; *δ*_H_ (CDCl_3_) 1.22 (9H, s, Me_3_C), 2.24 (3H, s, CH_3_CO), 6.94 (1H, s, thiazole 5-H), 7.14 (1H, dd, aryl H), 7.26 (1H, t, ArH), 7.49 (1H, t, ArH) and 7.63 (1H, dd, ArH); *δ*_C_ (CDCl_3_) 20.9, 27.4, 85.8, 112.7, 123.3, 126.1, 127.7, 130.3, 133.3, 136.4, 148.6, 150.2, 157.6, 166.6 and 168.8.

### 2-((4-Bromothiazol-2-yl)(*tert*-butoxycarbonyl)carbamoyl)phenyl acetate 24

2-(*t*-Butoxycarbonyl)amino-4-bromothiazole 6 (0.264 g, 0.95 mmol) was dissolved in THF (5 mL) with Et_3_N (0.30 mL, 2.1 mmol) and treated with *O*-acetylsalicyloyl chloride 9 (0.35 g, 1.80 mmol) added portionwise at 20 °C with stirring. After 16 h, the reaction mixture was diluted with EtOAc (30 mL) and worked up for a neutral product, which was purified by chromatography, eluting with a gradient of 5 to 7.5% EtOAc in hexane to afford the title compound 24 (0.292 g, 70%). Found: *m*/*z* (ES +ve mode) 462.9937; C_17_H_17_BrN_2_O_5_SNa (MNa^+^) requires *m*/*z*, 462.9939; *δ*_H_ (CDCl_3_) 1.32 (9H, s, Me_3_C), 2.34 (3H, s, CH_3_CO), 7.19 (1H, s, thiazole 5-H), 7.24 (1H, d, Ar H), 7.36 (1H, t, Ar H), 7.58 (1H, t, Ar H) and 7.68 (1H, d, ArH); *δ*_C_ (CDCl_3_) 20.9, 27.4, 85.8, 116.5, 122.3, 123.2, 126.1, 127.8, 130.3, 133.2, 148.5, 150.2, 158.4, 166.6 and 168.8.

### Crystallographic methods

Single crystals of C_21_H_15_N_2_O_6_SCl 13, C_7_H_8_N_2_O_3_S 15 and C_5_H_5_ClN_2_OS 19 were submitted for X-ray structural determination. A suitable crystal was selected and mounted on a MiTeGen tip using parabol oil and centred on a XtaLAB AFC12 (RCD3): Kappa single diffractometer. The crystal was kept at 100.01(10) K during data collection. Using Olex2,^45^ the structure was solved with the ShelXT^46^ structure solution program using Intrinsic Phasing and refined with the ShelXL^47^ refinement package using Least Squares minimisation.

### Crystallographic data

Cif files for compounds 13, 15 and 19 have been deposited in the CCDC database, no. CCDC 2362657, CCDC 2330479 and CCDC 2330480, respectively. The full data files have been added to ESI.†

## Data availability

The data supporting this article have been included as part of the ESI.†

## Conflicts of interest

There are no conflicts of interest to declare.

## Supplementary Material

RA-014-D4RA04959D-s001

RA-014-D4RA04959D-s002
