# Supplementary material for: Convenient syntheses of 2-acylamino-4-halothiazoles and acylated derivatives using a versatile Boc-intermediate
Source: RSC Adv. 2024 Sep 2;14(38):27894–903. doi: 10.1039/d4ra04959d (PMC11367624; doi:10.1039/d4ra04959d)
Supplement: RA-014-D4RA04959D-s001 [file RA-014-D4RA04959D-s001.pdf]

Supplementary Information (SI) for RSC Advances.
This journal is © The Royal Society of Chemistry 2024

Convenient syntheses of 2-acylamino-4-halothiazoles and acylated derivatives using a versatile Boc-intermediate

Sophie Pate, Joshua Taujanskas, Robyn Wells, Craig M. Robertson, Paul M. O'Neill and Andrew V. Stachulski*

Department of Chemistry, University of Liverpool, Liverpool L69 7ZD, U.K.

Supporting Information

1. Photocopy 1H and 13C NMR spectra for compounds described,

2. Full Cif crystallographic data files for **13, 15** and **19**.

*

* To whom correspondence should be addressed: stachuls@liv.ac.uk or 0151-794-3482

1. Photocopy 1H (400 MHz) and 13C (100 MHz) NMR spectra for compounds described

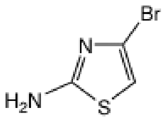

7

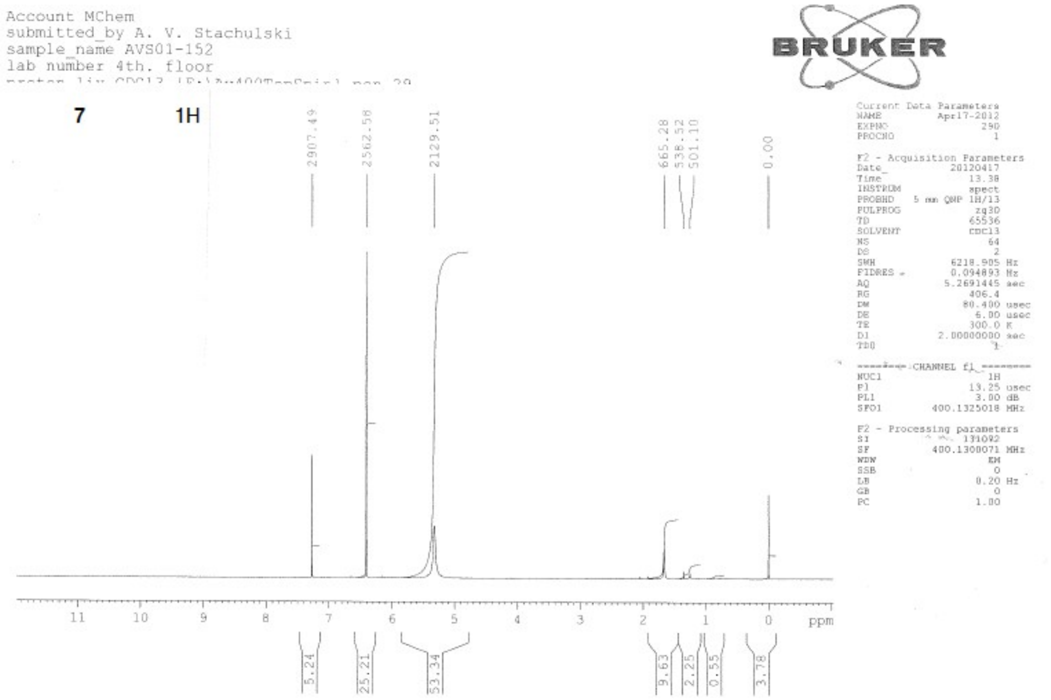

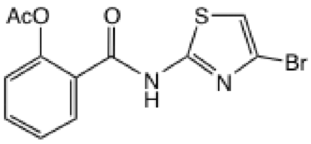

10

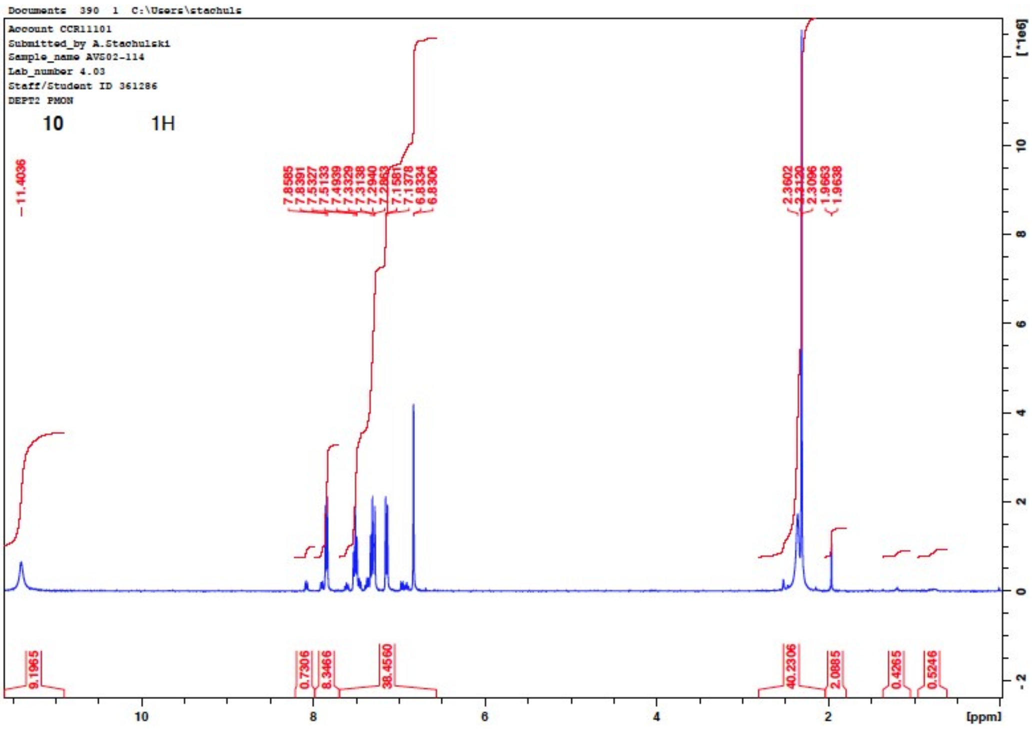

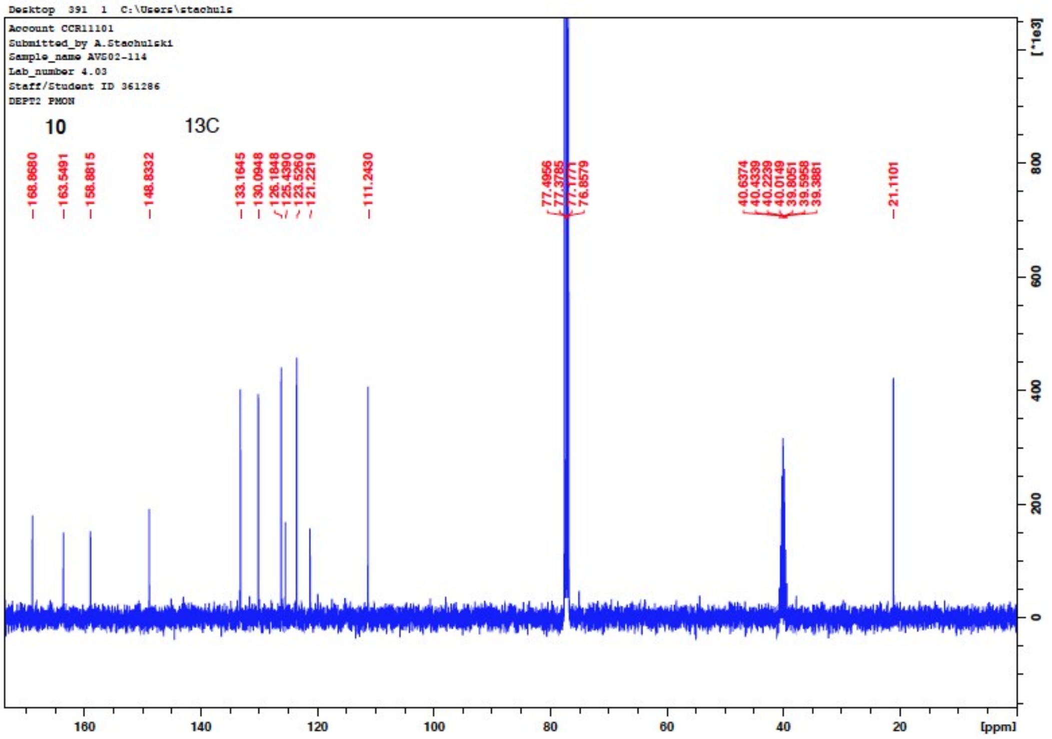

Contains a little d_6-DMSO for solubility.

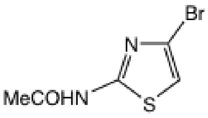

11

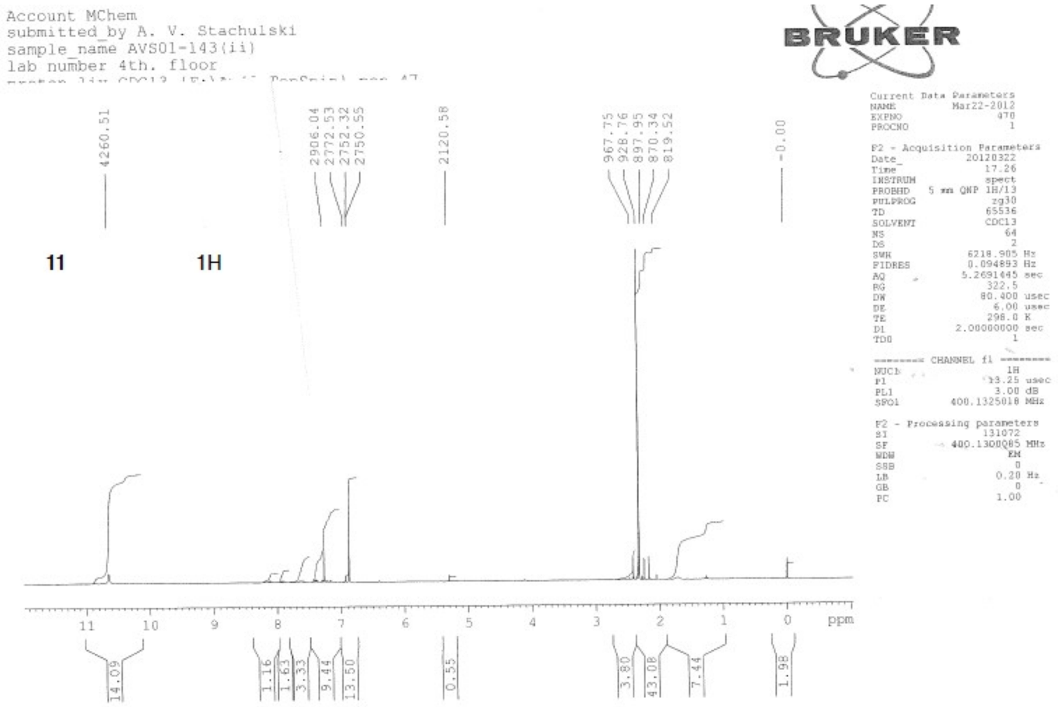

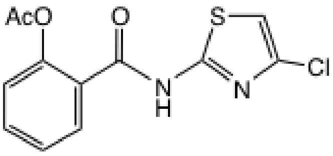
AcO
O
S
Cl
N
H
N

12

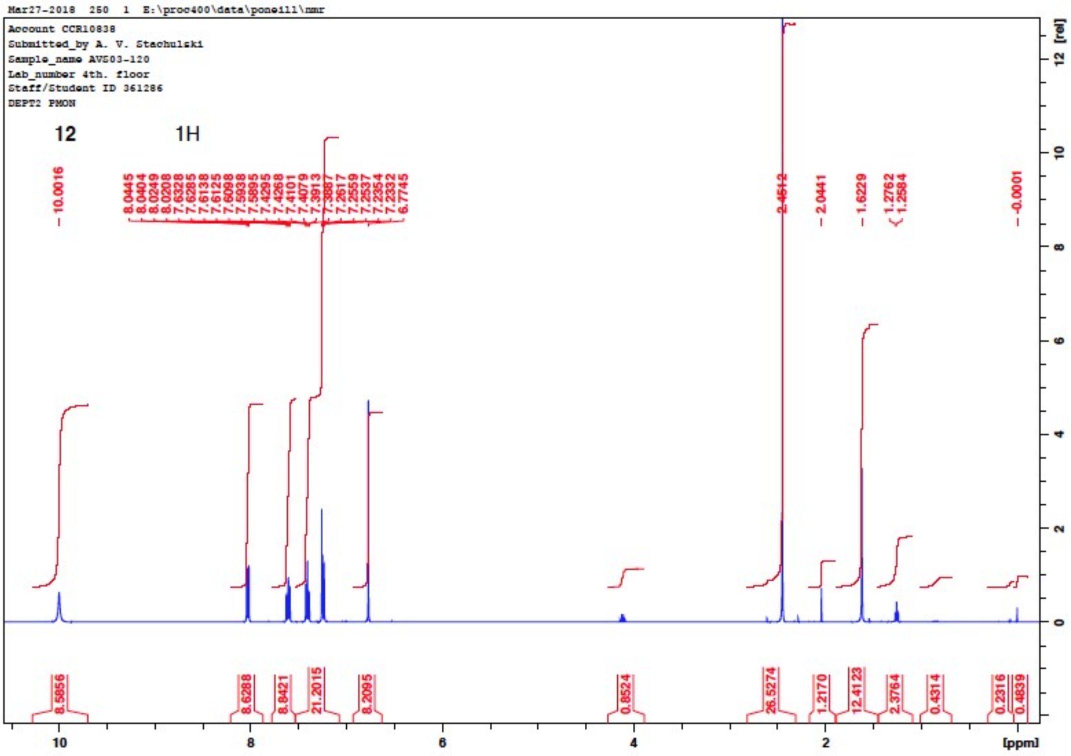
Mar27-2018 250 1 E:\proc400\data\poneill\nmr
Account CCR10838
Submitted_by A. V. Stachulski
Sample_name AVS03-120
Lab_number 4th. floor
Staff/Student ID 361286
DEPT2 PMON
12
1H
[ppm]
[rel]

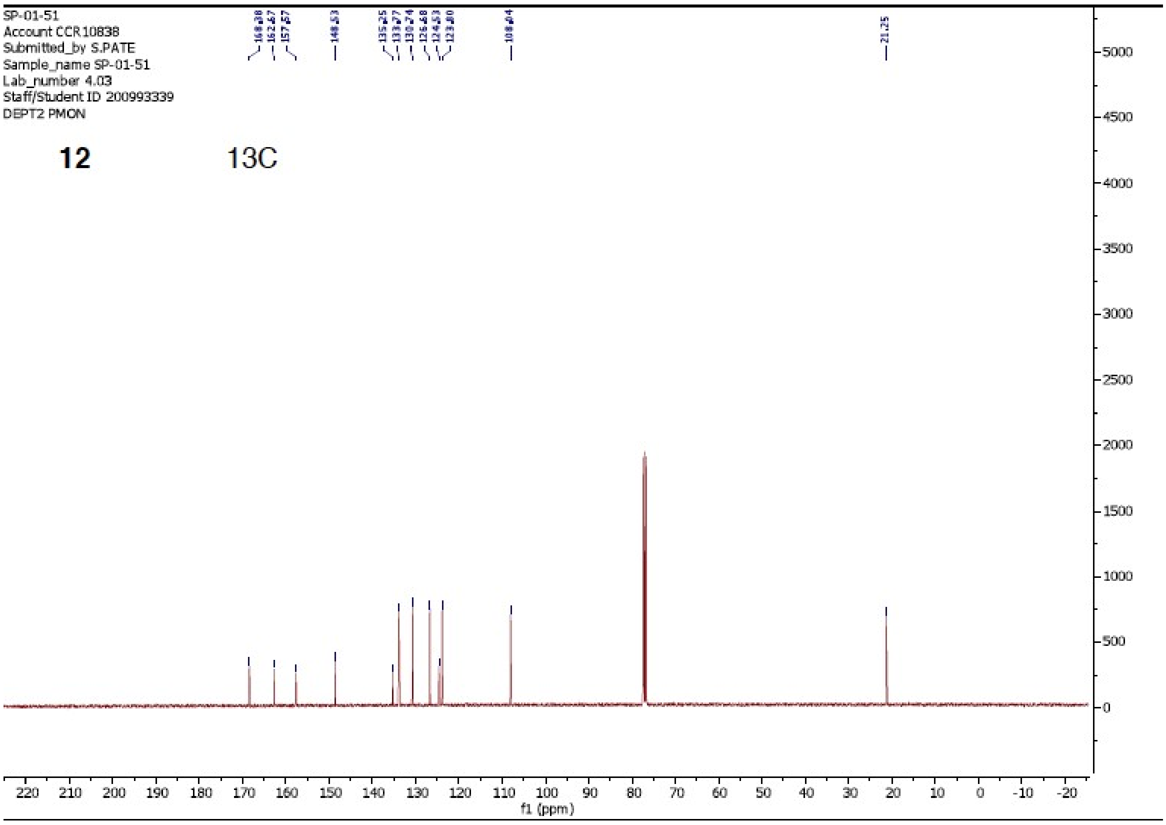
SP-01-51
Account CCR10838
Submitted_by S.PATE
Sample_name SP-01-51
Lab_number 4.03
Staff/Student ID 200993339
DEPT2 PMON
12
13C
f1 (ppm)

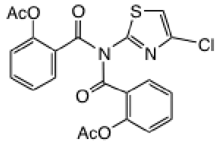

13

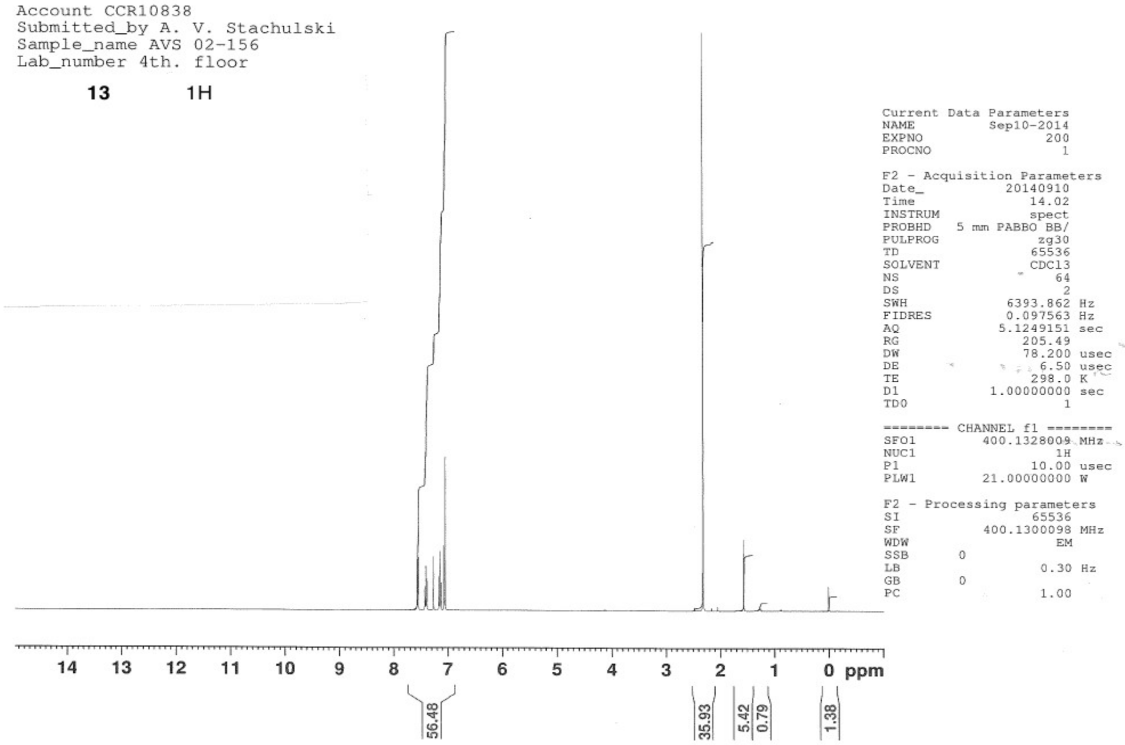

Account CCR10838
Submitted_by A. V. Stachulski
Sample_name AVS 02-156
Lab_number 4th. floor
13 1H
Current Data Parameters
NAME Sep10-2014
EXPNO 200
PROCNO 1
F2 - Acquisition Parameters
Date_ 20140910
Time 14.02
INSTRUM spect
PROBHD 5 mm PABBO BB/
PULPROG zg30
TD 65536
SOLVENT CDCl3
NS 64
DS 2
SWH 6393.862 Hz
FIDRES 0.097563 Hz
AQ 5.1249151 sec
RG 205.49
DW 78.200 usec
DE 6.50 usec
TE 298.0 K
D1 1.00000000 sec
TD0 1
======== CHANNEL f1 ========
SFO1 400.1328009 MHz
NUC1 1H
P1 10.00 usec
PLW1 21.00000000 W
F2 - Processing parameters
SI 65536
SF 400.1300098 MHz
WDW EM
SSB 0
LB 0.30 Hz
GB 0
PC 1.00
14 13 12 11 10 9 8 7 6 5 4 3 2 1 0 ppm
56.48
35.93
5.42
0.79
1.38

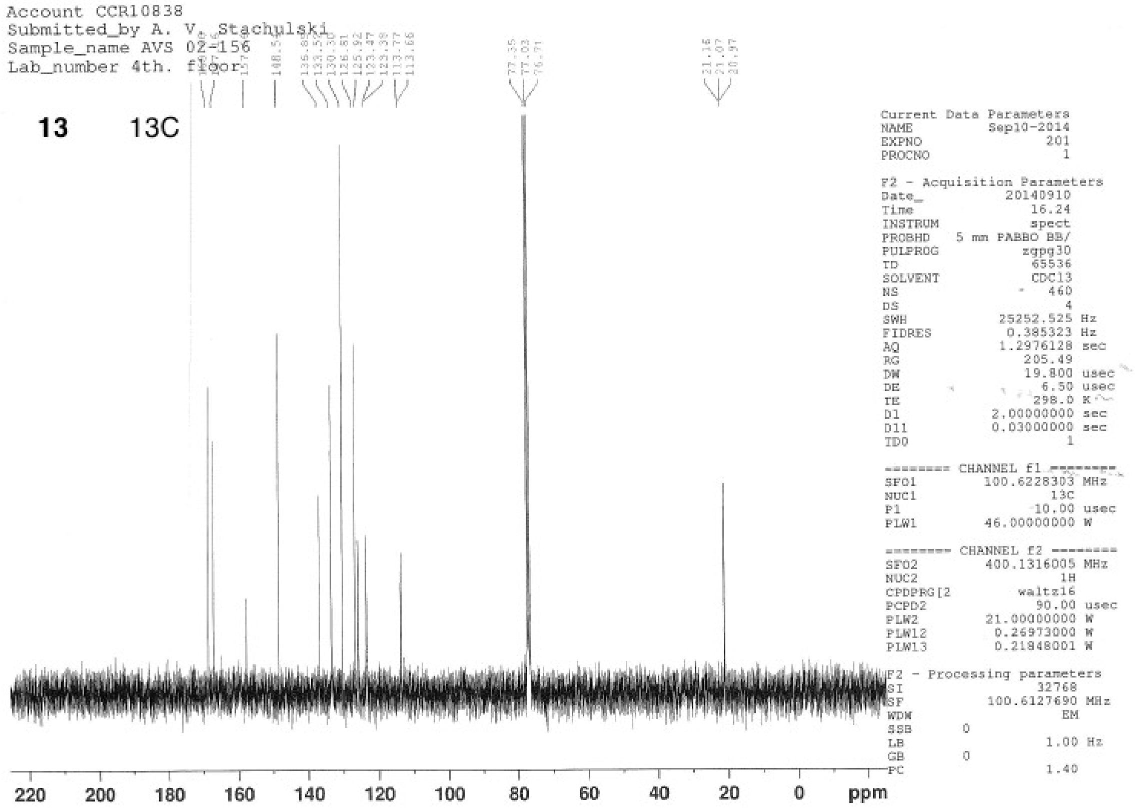

Account CCR10838
Submitted_by A. V. Stachulski
Sample_name AVS 02-156
Lab_number 4th. floor
77.35
77.03
76.71
21.16
21.07
20.97
13 13C
Current Data Parameters
NAME Sep10-2014
EXPNO 201
PROCNO 1
F2 - Acquisition Parameters
Date_ 20140910
Time 16.24
INSTRUM spect
PROBHD 5 mm PABBO BB/
PULPROG zgpg30
TD 65536
SOLVENT CDCl3
NS 460
DS 4
SWH 25252.525 Hz
FIDRES 0.385323 Hz
AQ 1.2976128 sec
RG 205.49
DW 19.800 usec
DE 6.50 usec
TE 298.0 K
D1 2.00000000 sec
D11 0.03000000 sec
TD0 1
======== CHANNEL f1 ========
SFO1 100.6228303 MHz
NUC1 13C
P1 10.00 usec
PLW1 46.00000000 W
======== CHANNEL f2 ========
SFO2 400.1316005 MHz
NUC2 1H
CPDPRG[2 waltz16
PCPD2 90.00 usec
PLW2 21.00000000 W
PLW12 0.26973000 W
PLW13 0.21848001 W
F2 - Processing parameters
SI 32768
SF 100.6127690 MHz
WDW EM
SSB 0
LB 1.00 Hz
GB 0
PC 1.40
220 200 180 160 140 120 100 80 60 40 20 0 ppm

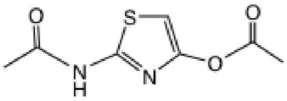

15

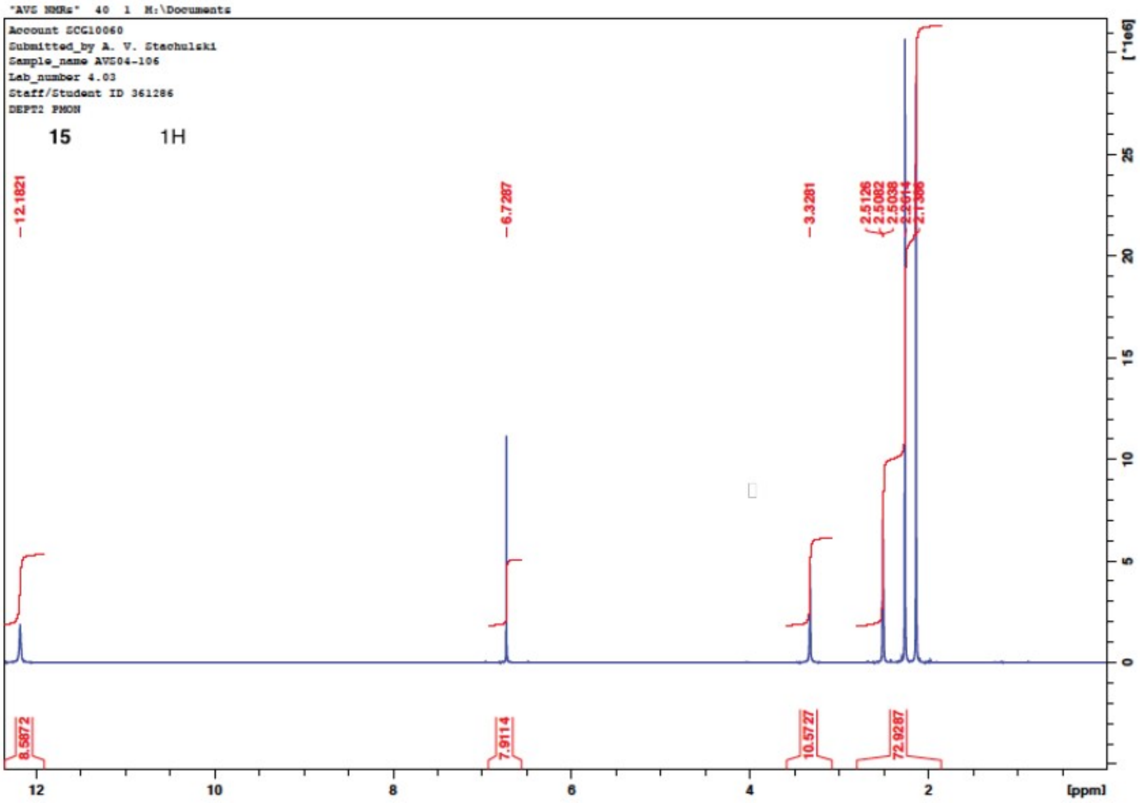
"AVS NMRs" 40 1 M:\Documents
Account SCG10060
Submitted_by A. V. Stachulski
Sample_name AVS04-106
Lab_number 4.03
Staff/Student ID 361286
DEPT2 PMON
15
1H
12.1821
6.7287
3.3281
2.5126
2.5082
2.5038
8.5872
7.9114
10.5727
72.9287
[ppm]
[*1e6]

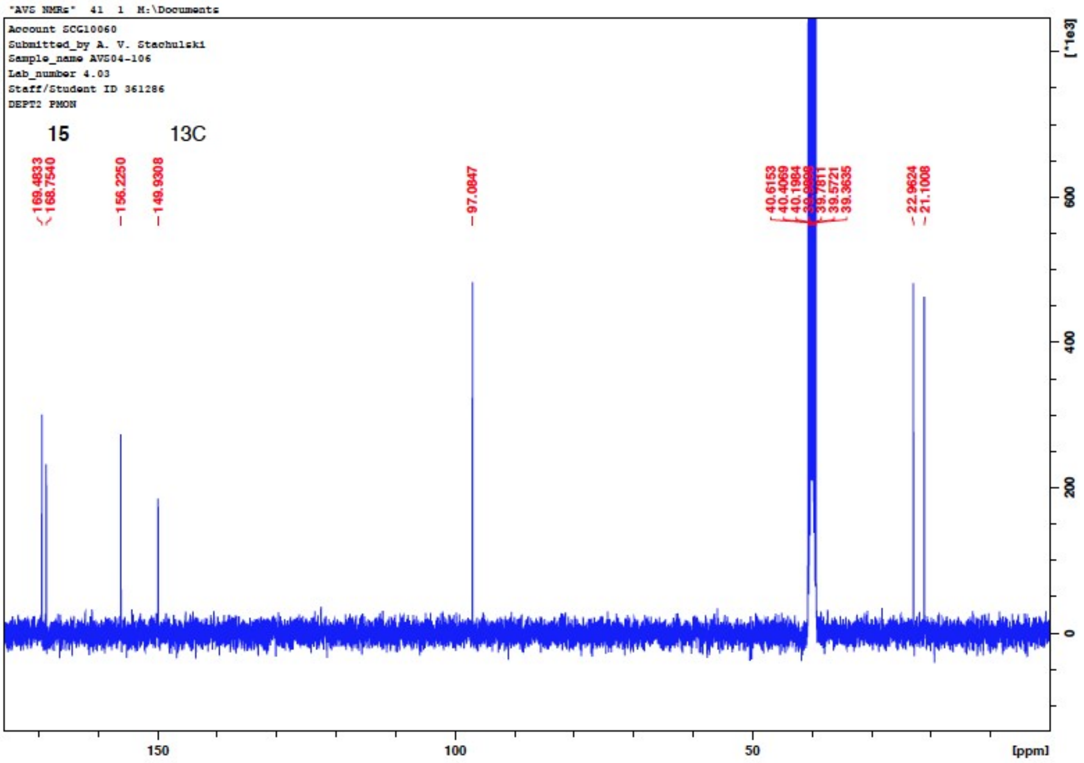
"AVS NMRs" 41 1 M:\Documents
Account SCG10060
Submitted_by A. V. Stachulski
Sample_name AVS04-106
Lab_number 4.03
Staff/Student ID 361286
DEPT2 PMON
15
13C
169.4833
168.7540
156.2250
149.9308
97.0847
40.6153
40.4069
40.1984
39.5721
39.3635
22.9624
21.1008
[ppm]
[*1e3]

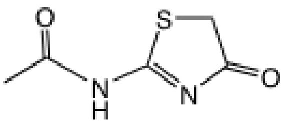

16

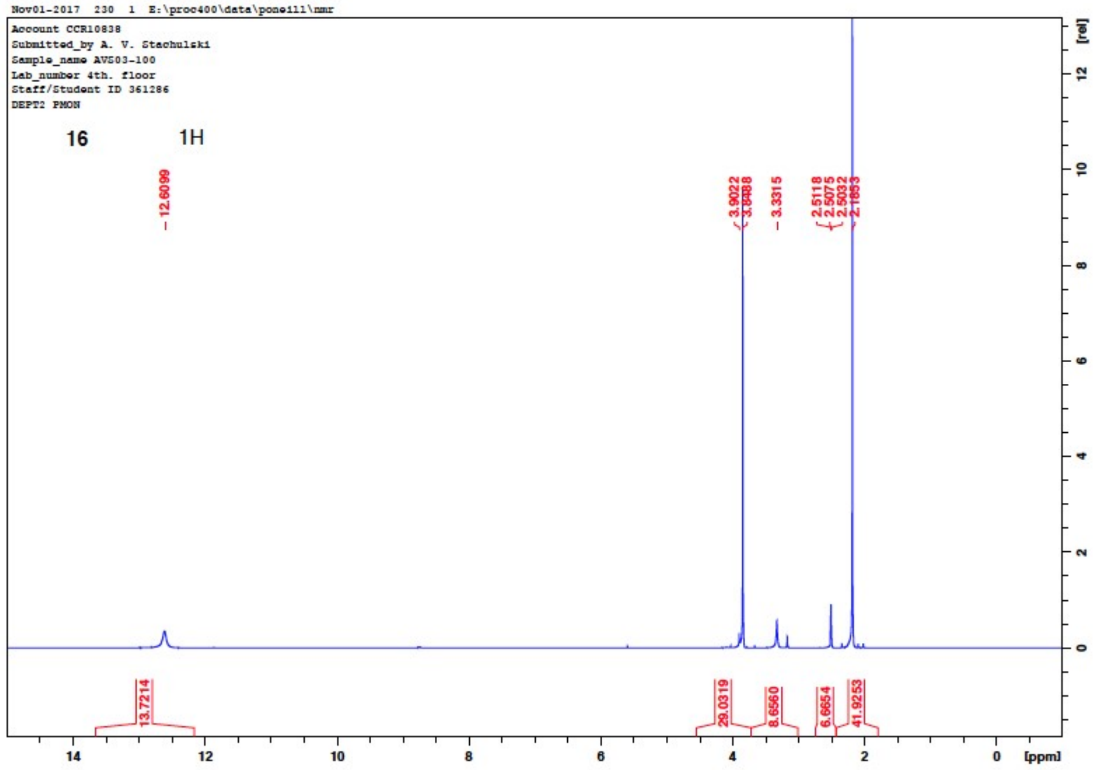
Nov01-2017 230 1 E:\proc400\data\poneill\nmr
Account CCR10838
Submitted_by A. V. Stachulski
Sample_name AVS03-100
Lab_number 4th. floor
Staff/Student ID 361286
DEPT2 PMON
16
1H
12.6099
3.9022
3.8488
3.3315
2.5118
2.5075
2.5032
2.1853
13.7214
29.0319
8.6560
6.6654
41.9253
[ppm]
[rel]

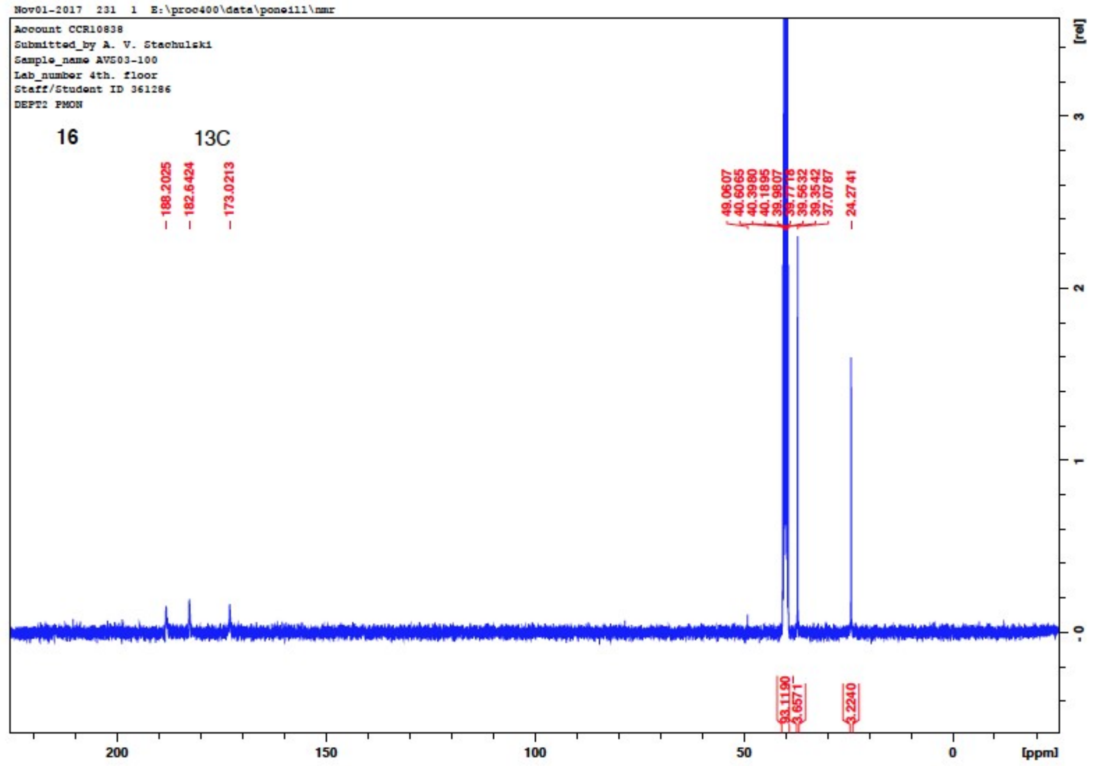
Nov01-2017 231 1 E:\proc400\data\poneill\nmr
Account CCR10838
Submitted_by A. V. Stachulski
Sample_name AVS03-100
Lab_number 4th. floor
Staff/Student ID 361286
DEPT2 PMON
16
13C
188.2025
182.6424
173.0213
49.0607
40.6065
40.3980
40.1895
39.9807
39.7718
39.5632
39.3542
37.0787
24.2741
93.1190
3.6571
3.2240
[ppm]
[rel]

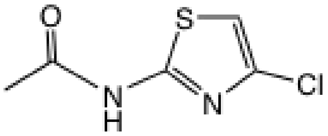

17

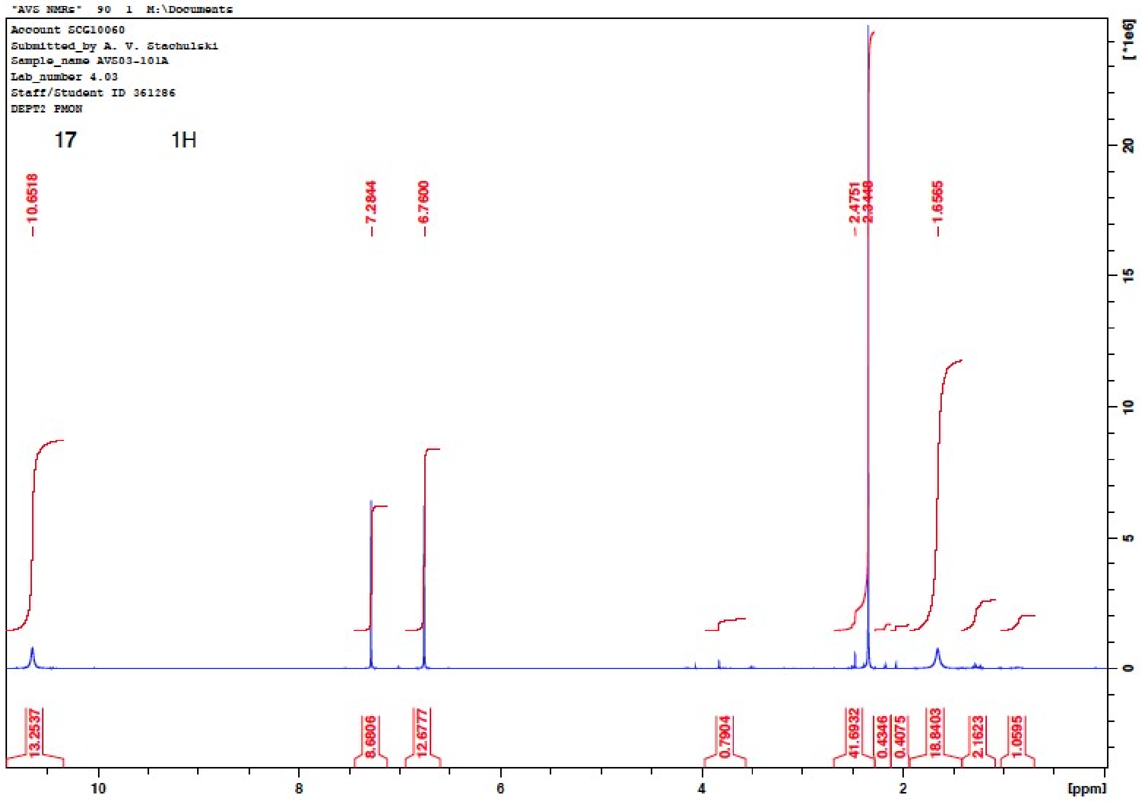

"AVS NMRs" 90 1 M:\Documents
Account SCG10060
Submitted_by A. V. Stachulski
Sample_name AVS03-101A
Lab_number 4.03
Staff/Student ID 361286
DEPT2 PMON
17
1H
10.6518
7.2844
6.7600
2.4751
1.6565
13.2537
8.6806
12.6777
0.7904
41.6932
0.4346
0.4075
18.8403
2.1623
1.0595
[ppm]
[*1e6]

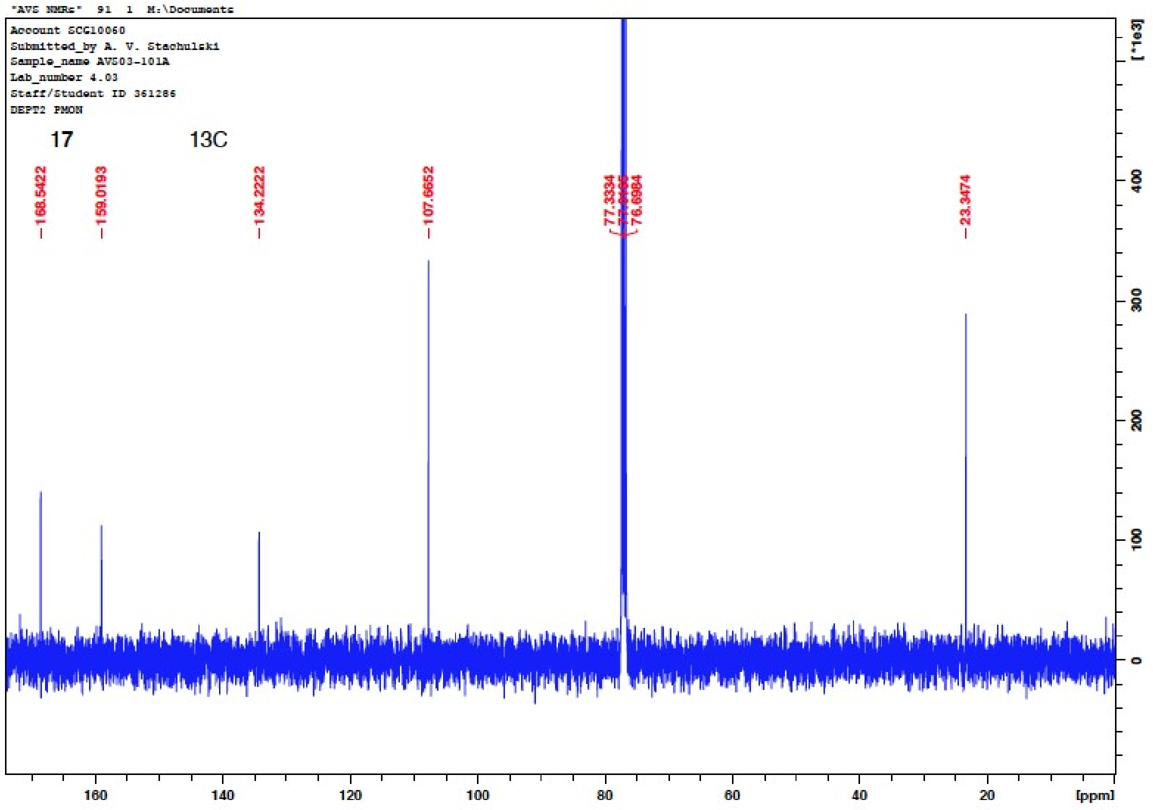

"AVS NMRs" 91 1 M:\Documents
Account SCG10060
Submitted_by A. V. Stachulski
Sample_name AVS03-101A
Lab_number 4.03
Staff/Student ID 361286
DEPT2 PMON
17
13C
168.5422
159.0193
134.2222
107.6652
77.3334
76.6984
23.3474
[ppm]
[*1e3]

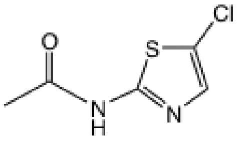

19

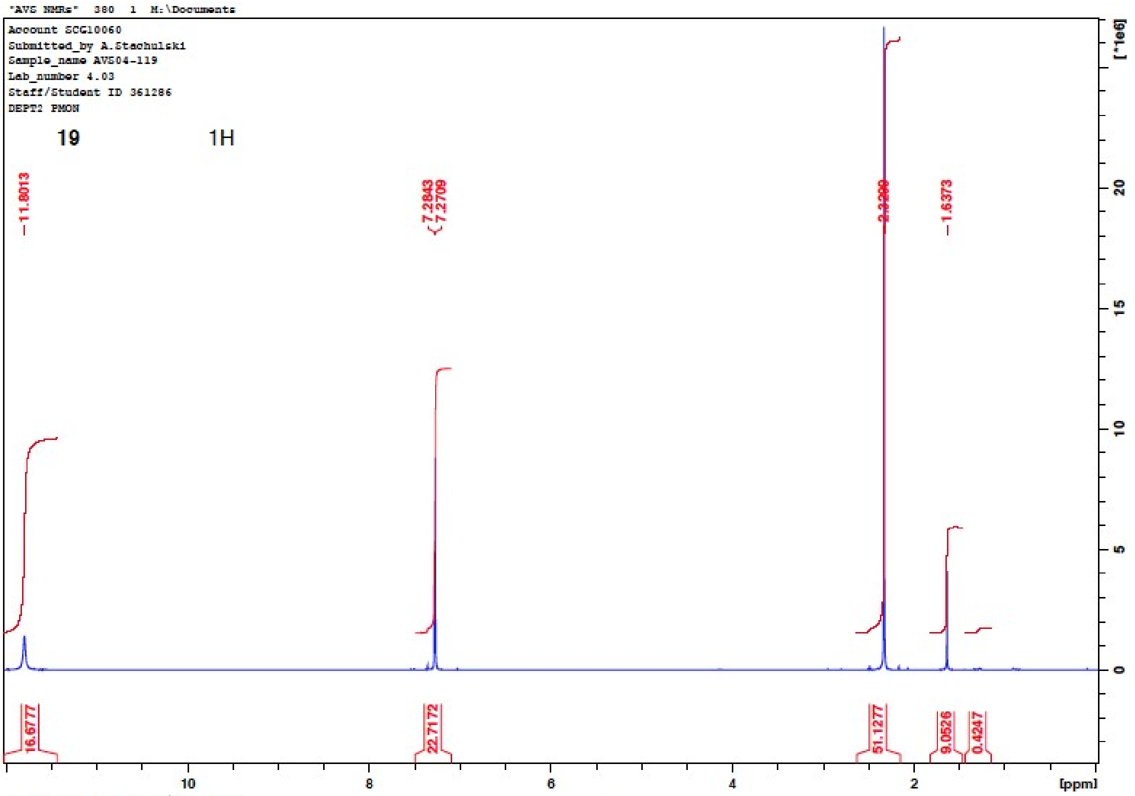
"AVS NMRs" 380 1 M:\Documents
Account SCG10060
Submitted_by A.Stachulski
Sample_name AVS04-119
Lab_number 4.03
Staff/Student ID 361286
DEPT2 PMON
19
1H
11.8013
7.2843
7.2709
2.3299
1.6373
16.6777
22.7172
51.1277
9.0526
0.4247
[ppm]
[*1e6]

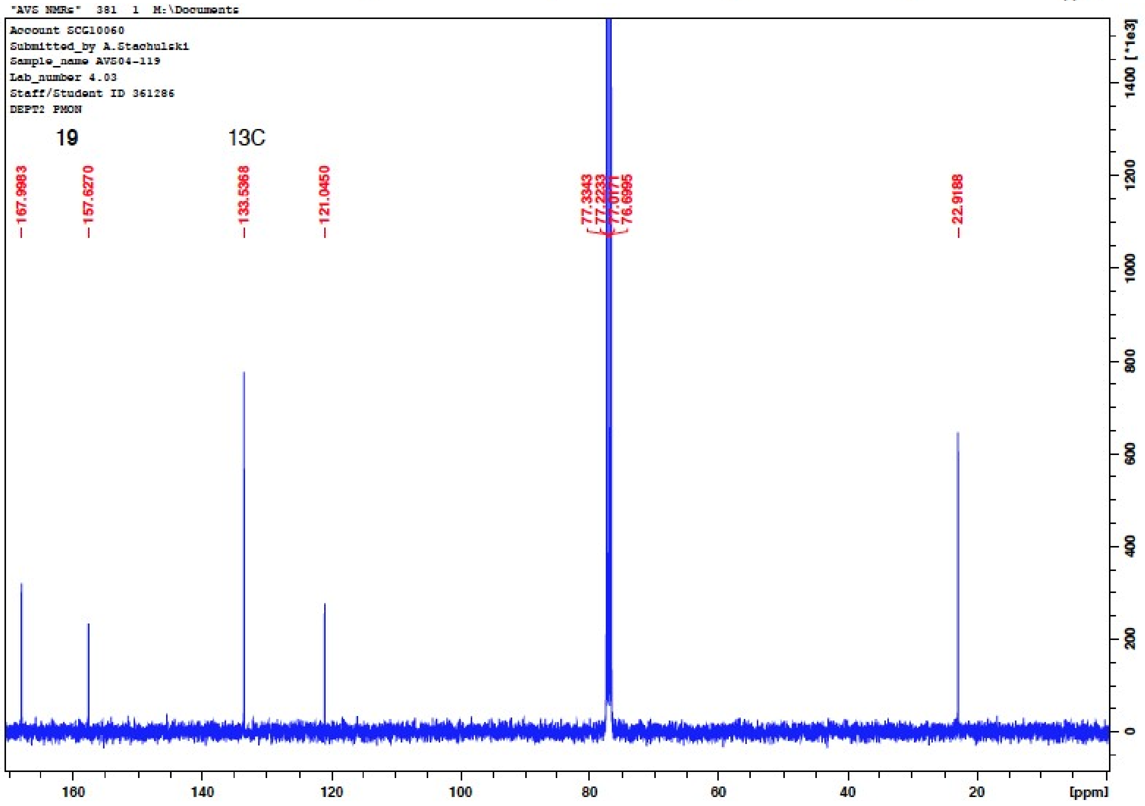
"AVS NMRs" 381 1 M:\Documents
Account SCG10060
Submitted_by A.Stachulski
Sample_name AVS04-119
Lab_number 4.03
Staff/Student ID 361286
DEPT2 PMON
19
13C
167.9983
157.6270
133.5368
121.0450
77.3343
77.2233
76.6995
22.9188
[ppm]
[*1e3]

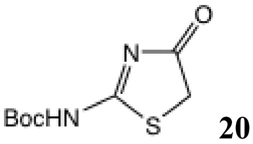

20

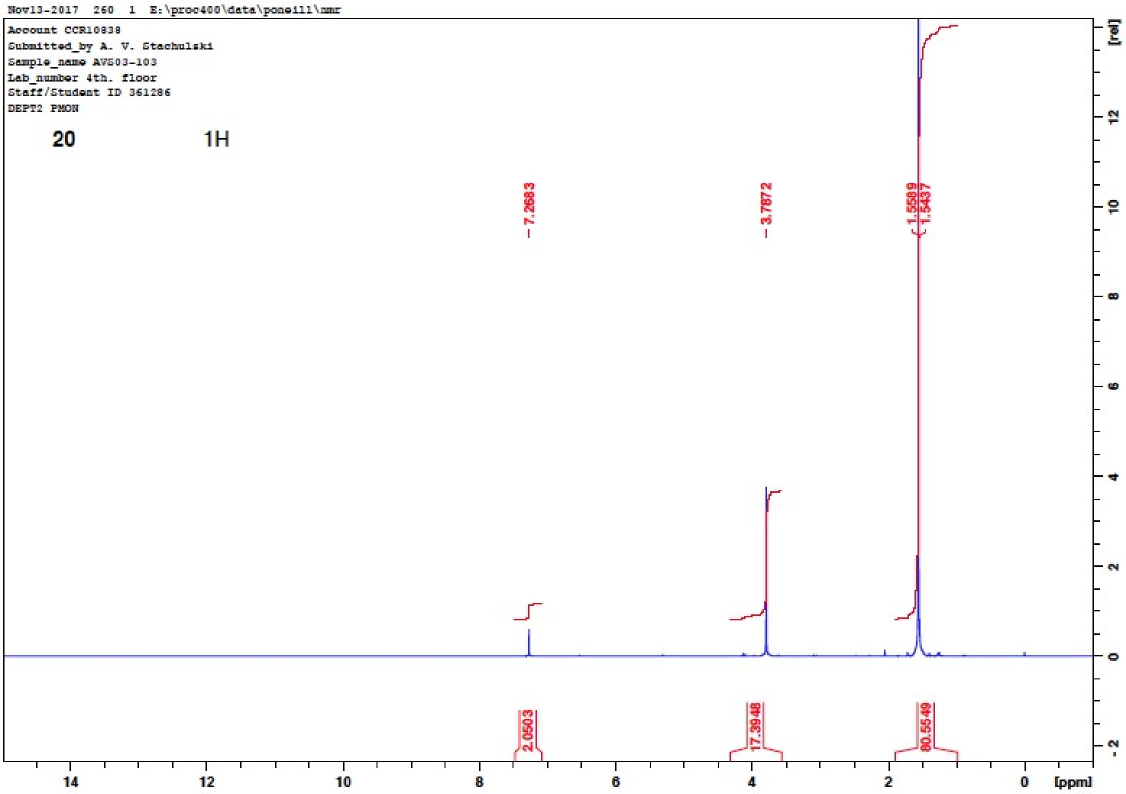
Nov13-2017 260 1 E:\proc400\data\poneill\nmr
Account CCR10838
Submitted_by A. V. Stachulski
Sample_name AVS03-103
Lab_number 4th. floor
Staff/Student ID 361286
DEPT2 PMON
20
1H
7.2683
3.7872
1.5589
1.5437
2.0503
17.3948
80.5549
[ppm]
[rel]

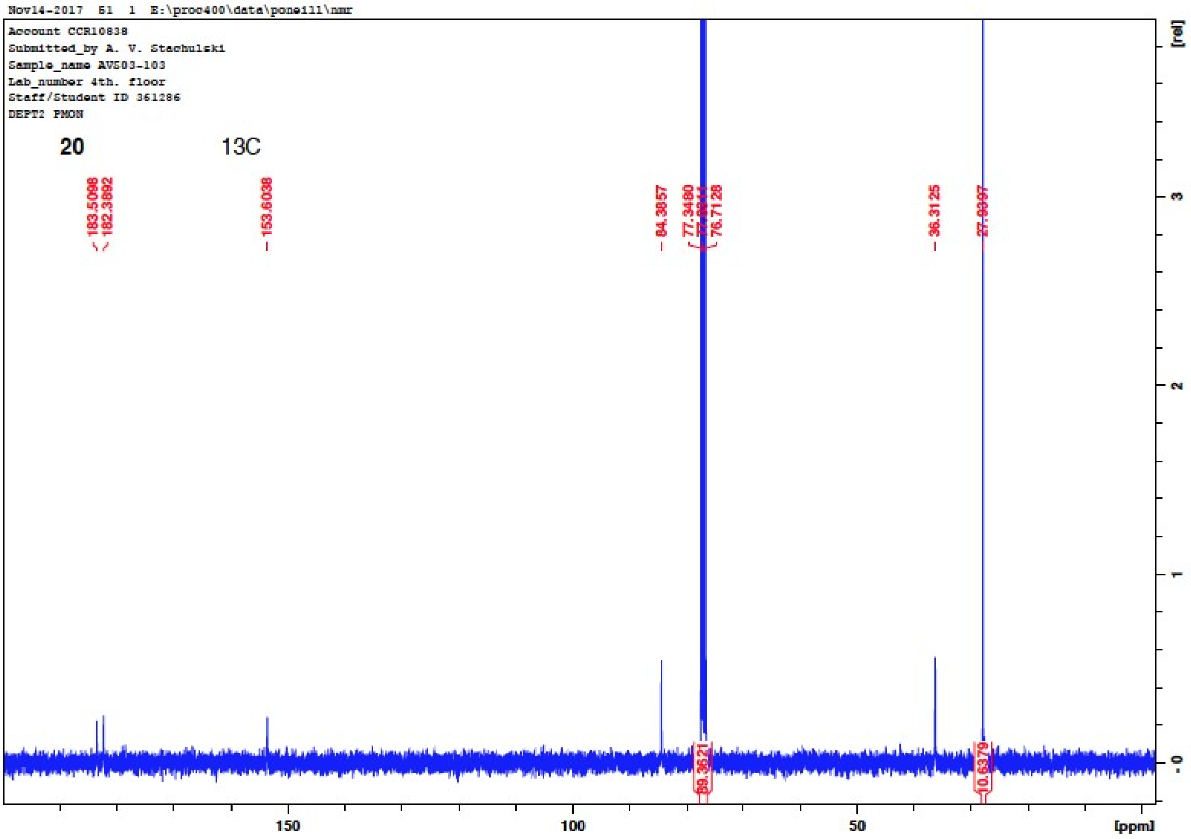
Nov14-2017 51 1 E:\proc400\data\poneill\nmr
Account CCR10838
Submitted_by A. V. Stachulski
Sample_name AVS03-103
Lab_number 4th. floor
Staff/Student ID 361286
DEPT2 PMON
20
13C
183.5098
182.3892
153.6038
84.3857
77.3480
76.7128
36.3125
[ppm]
[rel]

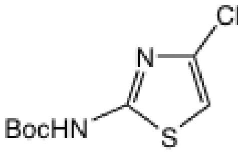
Cl
N
BocHN
S

21

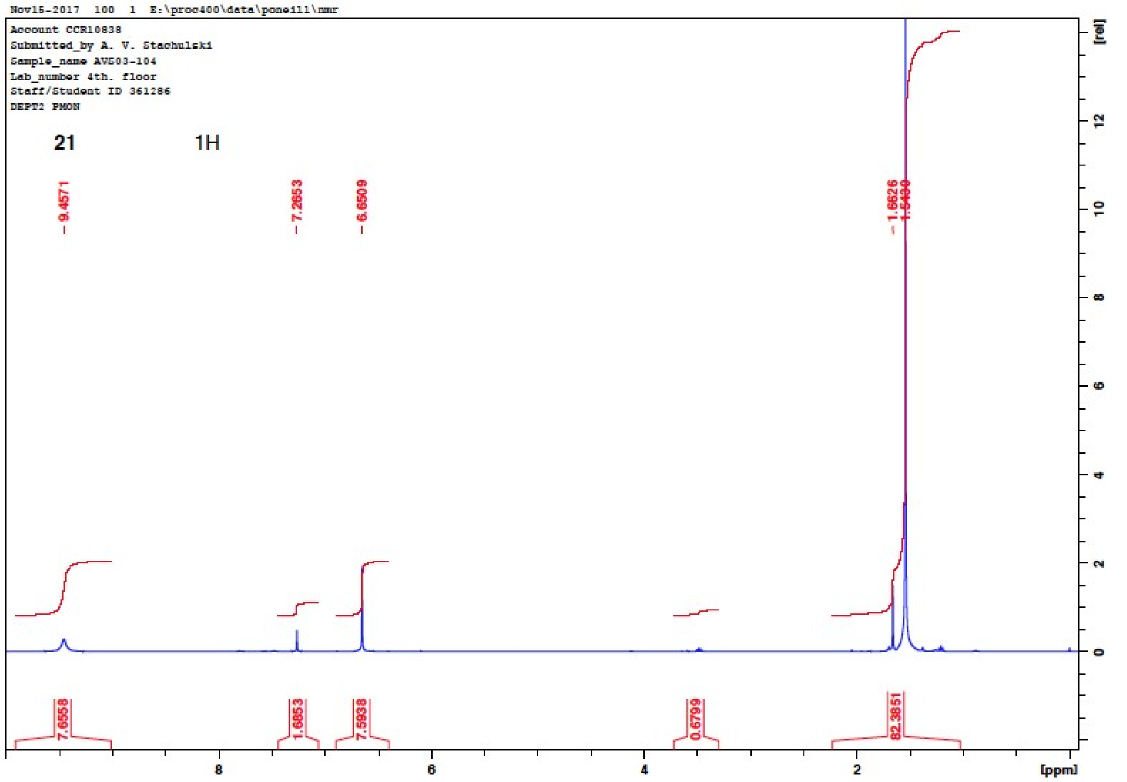
Nov15-2017 100 1 E:\proc400\data\poneill\nmr
Account CCR10838
Submitted_by A. V. Stachulski
Sample_name AVS03-104
Lab_number 4th. floor
Staff/Student ID 361286
DEPT2 PMON
21
1H
9.4571
7.2653
6.6509
1.6626
1.5430
7.6558
1.6853
7.5938
0.6799
82.3851
[rel]
[ppm]

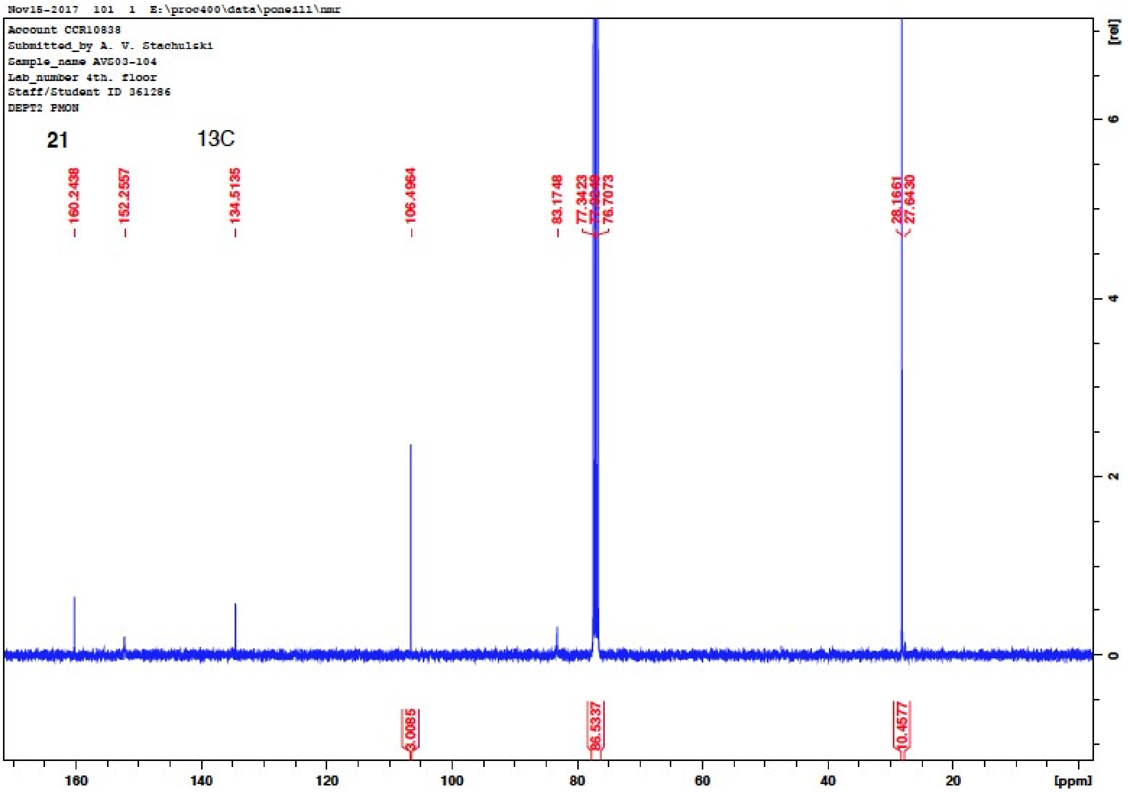
Nov15-2017 101 1 E:\proc400\data\poneill\nmr
Account CCR10838
Submitted_by A. V. Stachulski
Sample_name AVS03-104
Lab_number 4th. floor
Staff/Student ID 361286
DEPT2 PMON
21
13C
160.2438
152.2557
134.5135
106.4964
83.1748
77.3423
76.7073
28.1661
27.6430
3.0085
86.5337
10.4577
[rel]
[ppm]

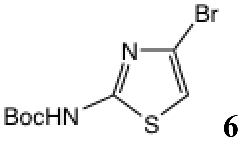
Br
N
BocHN
S

6

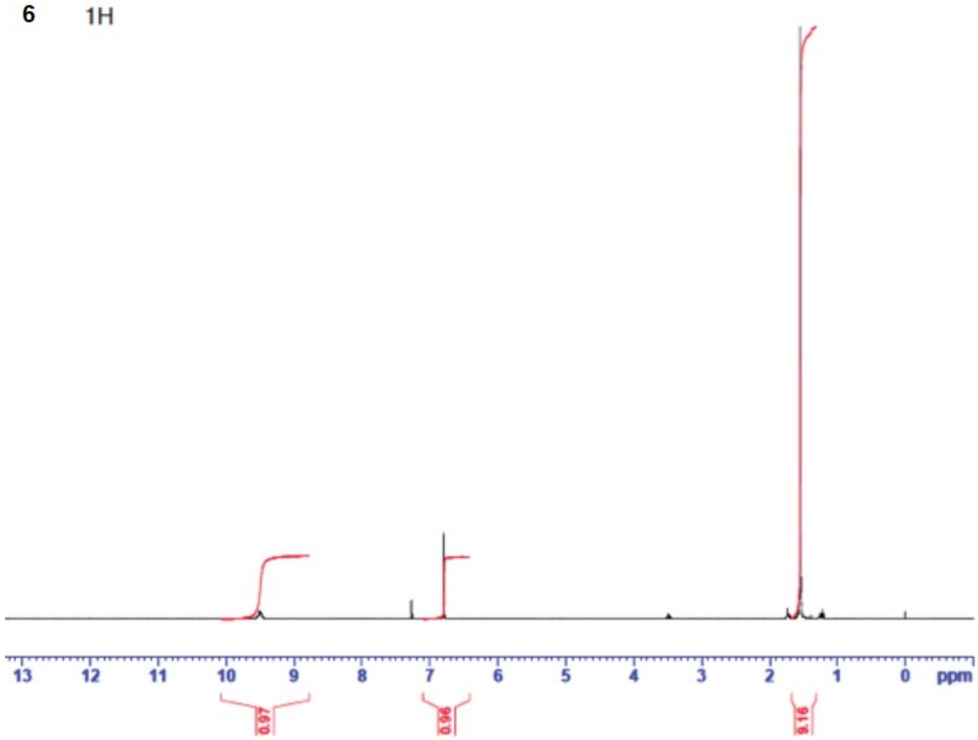
6 1H
13
12
11
10
9
8
7
6
5
4
3
2
1
0
ppm
0.97
0.96
9.16

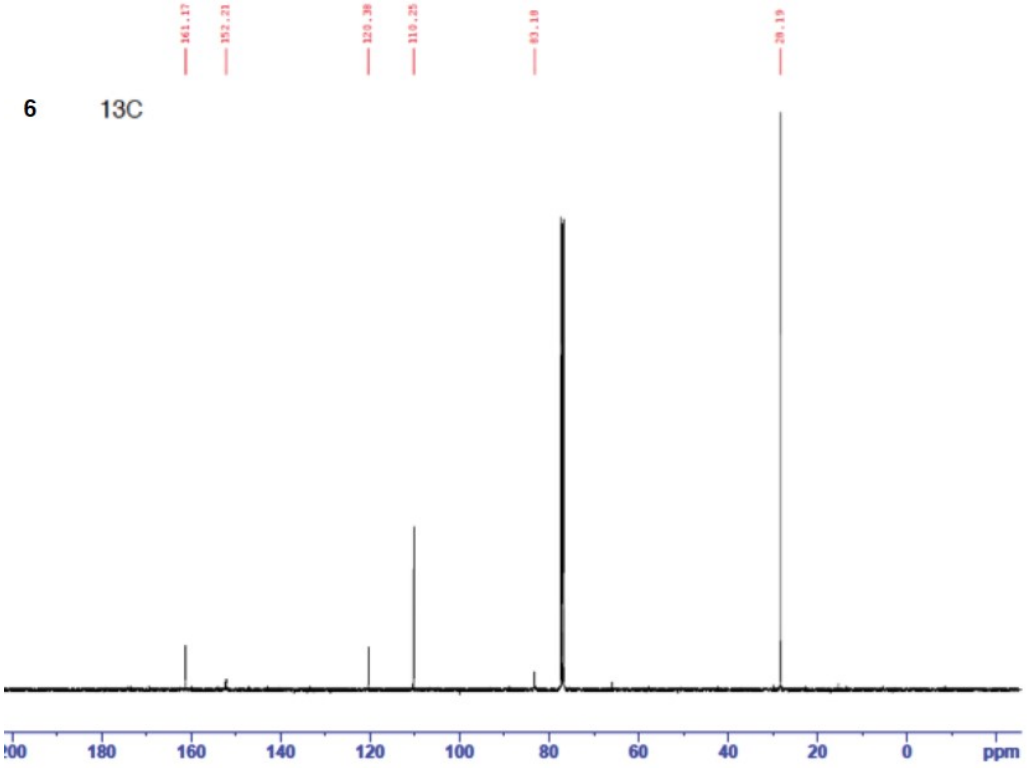
161.17
152.21
120.38
110.25
83.18
28.19
6 13C
180
160
140
120
100
80
60
40
20
0
ppm

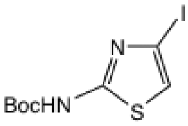
I
N
BocHN
S

22

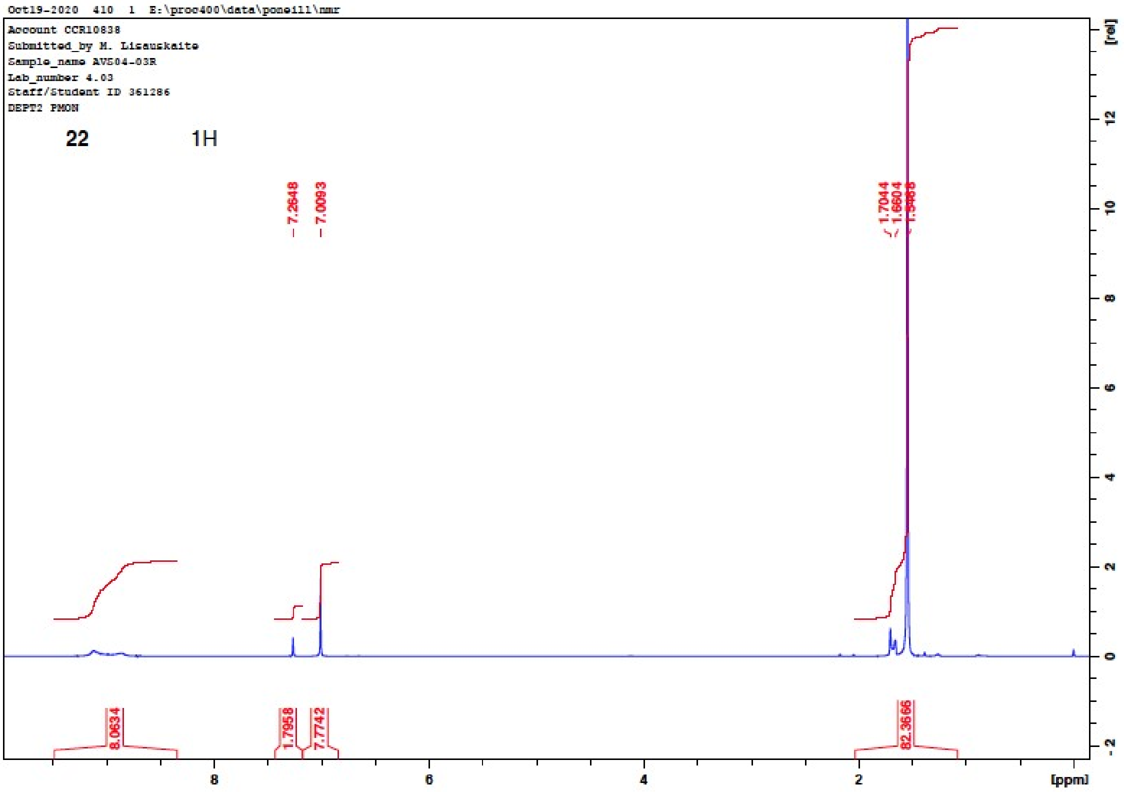
Oct19-2020 410 1 E:\proc400\data\poneill\nmr
Account CCR10838
Submitted_by M. Lisauskaite
Sample_name AV504-03R
Lab_number 4.03
Staff/Student ID 361286
DEPT2 PMON
22
1H
7.2648
7.0093
1.7044
1.6604
8.0634
1.7958
7.7742
82.3666
[ppm]
[rel]

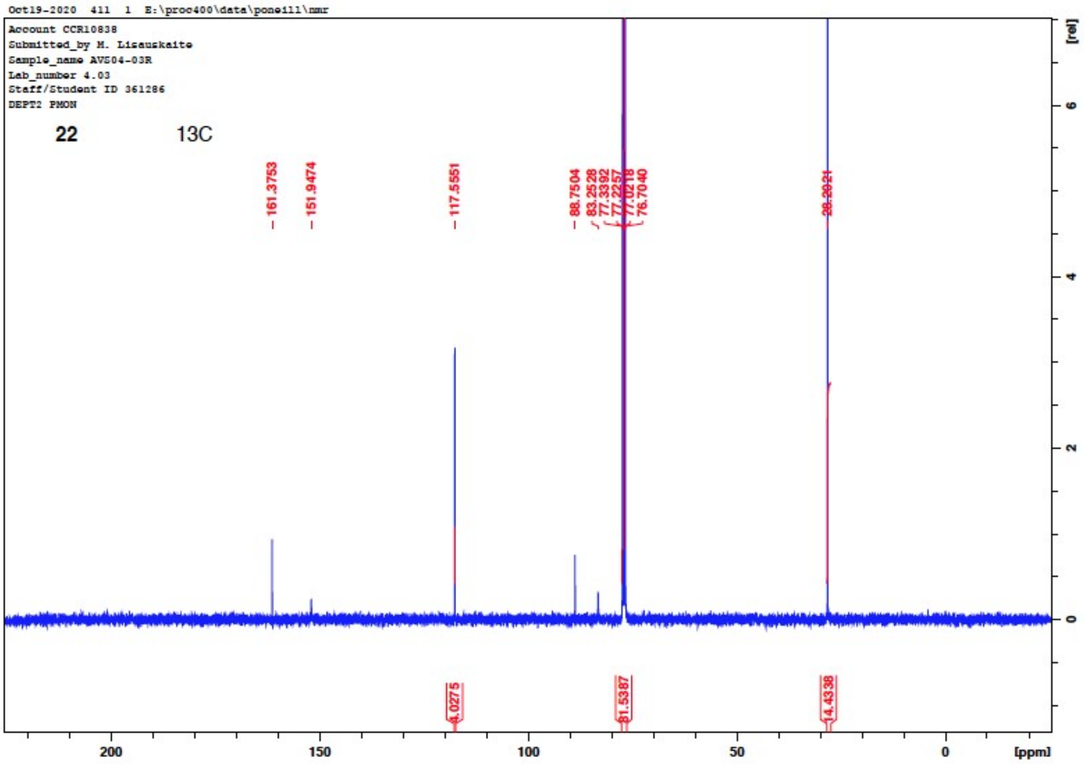
Oct19-2020 411 1 E:\proc400\data\poneill\nmr
Account CCR10838
Submitted_by M. Lisauskaite
Sample_name AV504-03R
Lab_number 4.03
Staff/Student ID 361286
DEPT2 PMON
22
13C
161.3753
151.9474
117.5551
88.7504
83.2528
77.3392
77.0218
76.7040
28.2021
[ppm]
[rel]

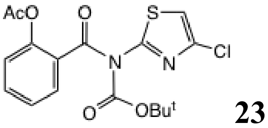
AcO
O
S
N
N
Cl
O
OBu^t

23

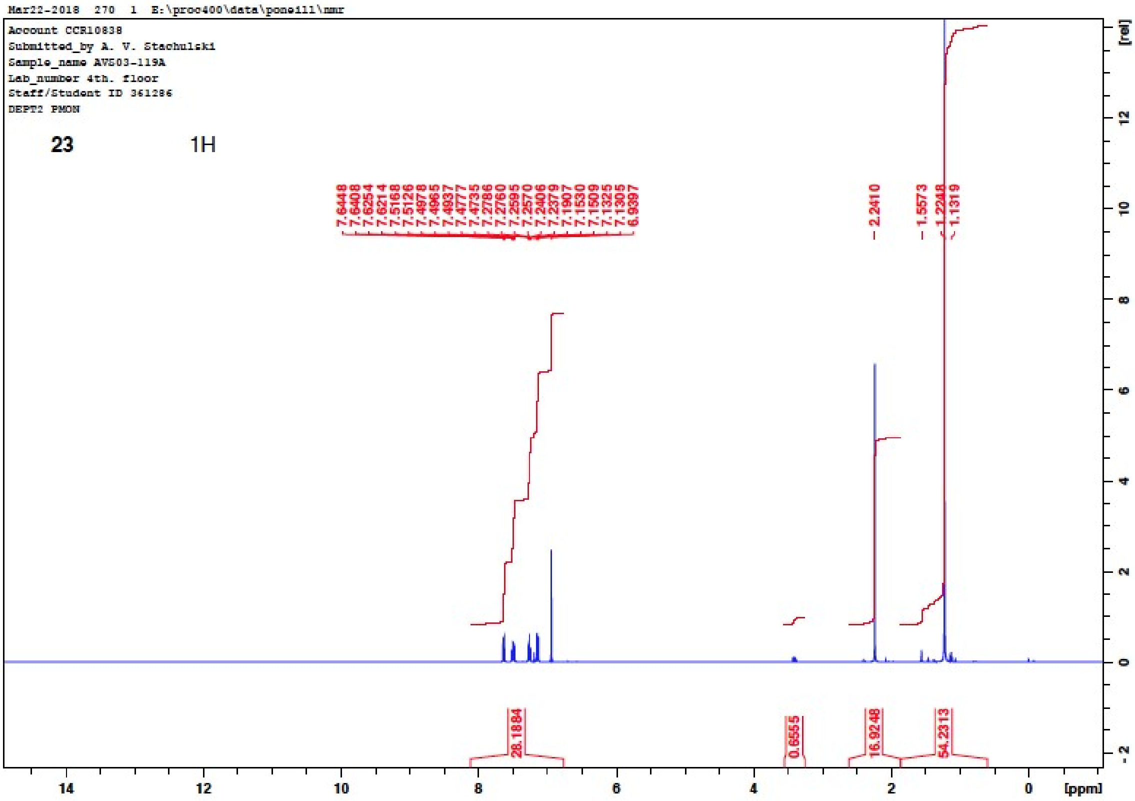
Mar22-2018 270 1 E:\proc400\data\poneill\nmr
Account CCR10838
Submitted_by A. V. Stachulski
Sample_name AVS03-119A
Lab_number 4th. floor
Staff/Student ID 361286
DEPT2 PMON
23
1H
7.6448
7.6408
7.6254
7.6214
7.5168
7.5126
7.4978
7.4965
7.4937
7.4777
7.4735
7.2786
7.2760
7.2595
7.2570
7.2406
7.2379
7.1907
7.1530
7.1509
7.1325
7.1305
6.9397
2.2410
1.5573
1.2248
1.1319
28.1884
0.6555
16.9248
54.2313
[rel]
[ppm]

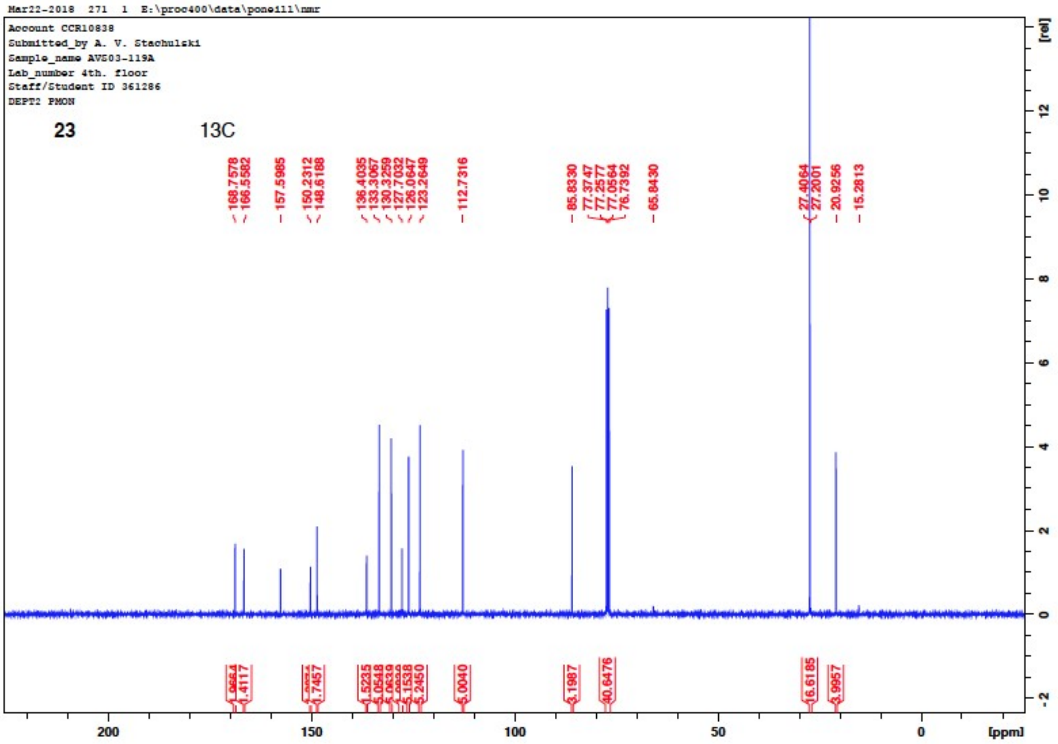
Mar22-2018 271 1 E:\proc400\data\poneill\nmr
Account CCR10838
Submitted_by A. V. Stachulski
Sample_name AVS03-119A
Lab_number 4th. floor
Staff/Student ID 361286
DEPT2 PMON
23
13C
168.7578
166.5582
157.5985
150.2312
148.6188
136.4035
133.3067
130.3259
127.7032
126.0647
123.2649
112.7316
85.8330
77.3747
77.2577
77.0564
76.7392
65.8430
27.4064
27.2001
20.9256
15.2813
6.0040
40.6476
[rel]
[ppm]

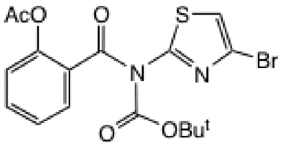
AcO
O
S
N
N
Br
O
OBut

24

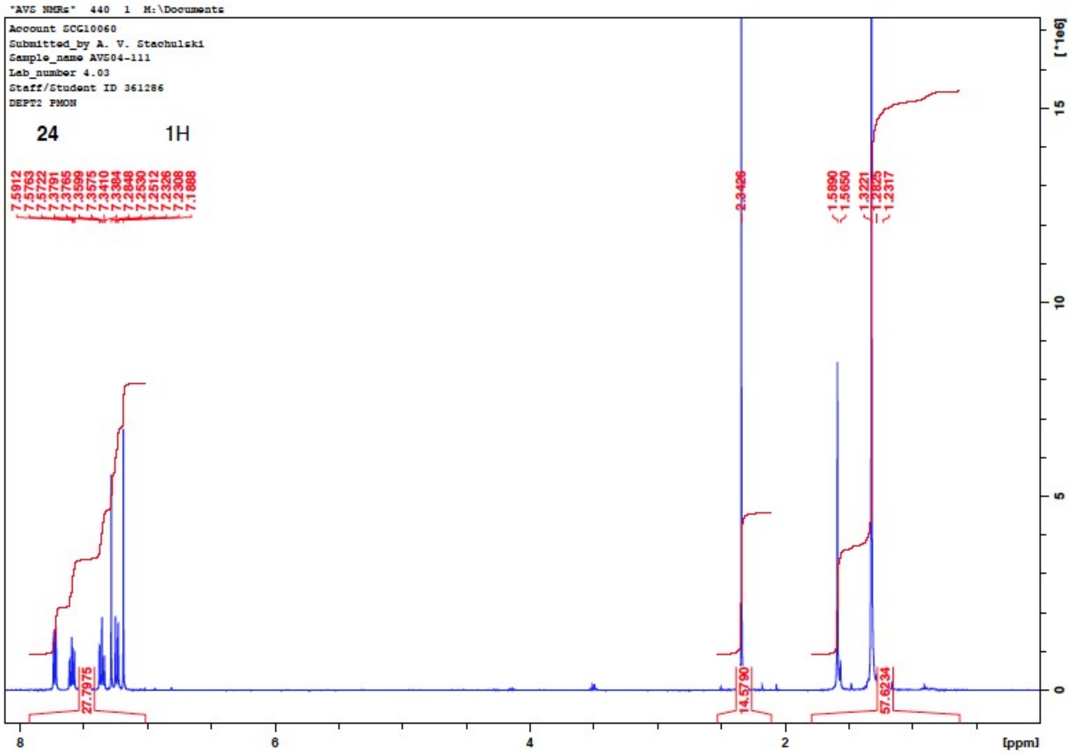
"AVS NMRs" 440 1 M:\Documents
Account SCG10060
Submitted_by A. V. Stachulski
Sample_name AVS04-111
Lab_number 4.03
Staff/Student ID 361286
DEPT2 PMON
24
1H
7.5912
7.5763
7.5722
7.3791
7.3765
7.3599
7.3575
7.3410
7.3384
7.2848
7.2530
7.2512
7.2326
7.2308
7.1888
2.3426
1.5890
1.5650
1.3221
1.2825
1.2317
27.7975
14.5790
57.6234
[ppm]
[*1e6]

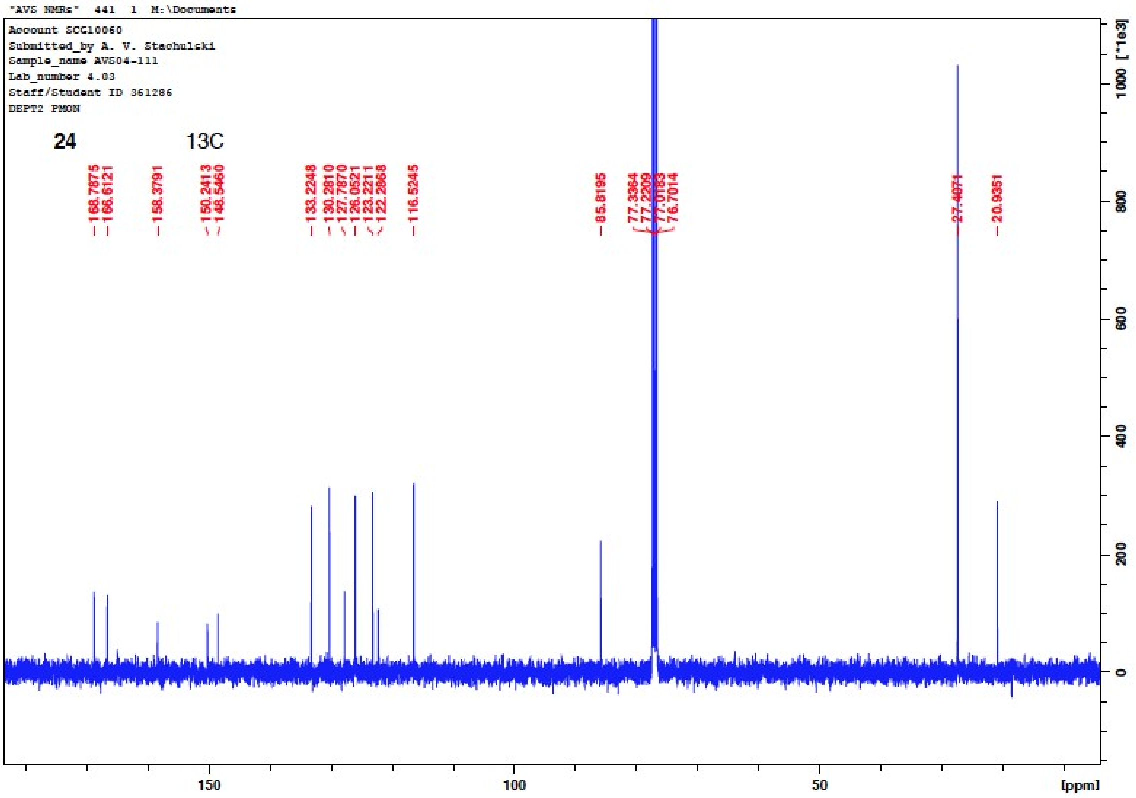
"AVS NMRs" 441 1 M:\Documents
Account SCG10060
Submitted_by A. V. Stachulski
Sample_name AVS04-111
Lab_number 4.03
Staff/Student ID 361286
DEPT2 PMON
24
13C
168.7875
166.6121
158.3791
150.2413
148.5460
133.2248
130.2810
127.7870
126.0521
123.2211
122.2868
116.5245
85.8195
77.3364
77.2209
77.0183
76.7014
27.4071
20.9351
[ppm]
[*1e3]

2. Full Cif crystallographic data files

Compound 13 CCDC 2362657.

Table 1 Crystal data and structure refinement for **13**.

Identification code	AVS_02_156
Empirical formula	$C_{41}H_{30}Cl_2N_4O_{12}S_2$
Formula weight	917.72
Temperature/K	150.01(10)
Crystal system	orthorhombic
Space group	*Pna2(1)*
a/Å	20.4035(6)
b/Å	8.6054(3)
c/Å	23.5849(6)
α/°	90.00
β/°	90.00
γ/°	90.00
Volume/Å^3	4141.0(2)
Z	4
ρ_{calc}g/cm^3	1.472
μ/mm^{-1}	0.327
F(000)	1888.0
Crystal size/mm^3	0.2 × 0.02 × 0.02
Radiation	Mo Kα (λ = 0.71073)
2Θ range for data collection/°	5.14 to 52.8
Index ranges	$-25 \leq h \leq 25$, $-10 \leq k \leq 8$, $-29 \leq l \leq 29$
Reflections collected	18121
Independent reflections	6797 [R_{int} = 0.0471, R_{sigma} = 0.0375]
Data/restraints/parameters	561/0/7
Goodness-of-fit on F^2	1.042
Final R indexes [I>=2σ (I)]	R_1 = 0.0441, wR_2 = 0.0999
Final R indexes [all data]	R_1 = 0.0584, wR_2 = 0.1082
Largest diff. peak/hole / e Å^{-3}	0.68/-0.50

Compound 15 CCDC 2330479.

Table 1 Crystal data and structure refinement for **15**.

Identification code	AVS04_106_auto

Empirical formula	$C_7H_8N_2O_3S$
Formula weight	200.21
Temperature/K	100.01(10)
Crystal system	orthorhombic
Space group	Pbcn
a/Å	22.162(3)
b/Å	7.1809(10)
c/Å	10.8505(15)
α/°	90
β/°	90
γ/°	90
Volume/Å^3	1726.8(4)
Z	8
ρ_{calc}g/cm^3	1.540
μ/mm^{-1}	0.349
F(000)	832.0
Crystal size/mm^3	0.22 × 0.05 × 0.02
Radiation	Mo Kα (λ = 0.71073)
2Θ range for data collection/°	5.964 to 60.416
Index ranges	$-27 \leq h \leq 27$, $-7 \leq k \leq 8$, $-9 \leq l \leq 14$
Reflections collected	7410
Independent reflections	2080 [R_{int} = 0.0205, R_{sigma} = 0.0205]
Data/restraints/parameters	2080/0/120
Goodness-of-fit on F^2	1.064
Final R indexes [I>=2σ (I)]	R_1 = 0.0306, wR_2 = 0.0833
Final R indexes [all data]	R_1 = 0.0349, wR_2 = 0.0867
Largest diff. peak/hole / e Å^{-3}	0.32/-0.28

Table 2 Fractional Atomic Coordinates (×10^4) and Equivalent Isotropic Displacement Parameters (Å^2×10^3) for **15**. U_{eq} is defined as 1/3 of of the trace of the orthogonalised U_{IJ} tensor.

**Atom**	***x***	***y***	***z***	**U(eq)**
S1	4888.7(2)	2475.9(4)	5969.1(3)	15.20(12)
O1	3769.9(4)	1186.3(14)	5314.5(8)	19.9(2)
O2	6326.8(4)	3863.7(14)	4148.0(8)	18.1(2)
O3	6808.2(5)	4590.6(17)	5910.2(9)	27.5(3)
N2	4383.6(5)	1802.6(16)	3708.2(9)	14.5(2)
N1	5384.8(5)	2740.6(15)	3839.4(10)	14.1(2)

C6	6789.8(6)	4604.9(18)	4811.2(12)	17.3(3)
C4	3846.7(6)	1246.3(18)	4202.4(12)	15.0(3)
C2	5798.7(5)	3277.9(18)	4697.0(11)	14.5(3)
C7	7244.0(6)	5435(2)	3963.6(13)	20.8(3)
C5	3372.4(6)	725(2)	3281.7(12)	20.5(3)
C1	4877.4(5)	2310.4(17)	4391.9(13)	13.2(3)
C3	5628.6(6)	3229.5(19)	5891.3(11)	16.1(3)

Table 3 Anisotropic Displacement Parameters ($Å^2 \times 10^3$) for **15**. The Anisotropic displacement factor exponent takes the form: $-2\pi^2[h^2a^{*2}U_{11}+2hka^*b^*U_{12}+\ldots]$.

**Atom**	**U_{11}**	**U_{22}**	**U_{33}**	**U_{23}**	**U_{13}**	**U_{12}**
S1	14.51(19)	21.6(2)	9.54(17)	0.22(11)	1.14(10)	0.47(11)
O1	17.2(5)	28.9(5)	13.6(5)	3.0(4)	3.0(3)	-0.1(4)
O2	14.2(4)	26.9(5)	13.3(4)	-1.8(4)	0.5(3)	-5.0(4)
O3	21.4(5)	45.1(7)	15.9(5)	-0.7(4)	-3.6(4)	-8.1(4)
N2	12.6(5)	21.1(6)	9.7(5)	0.5(4)	0.3(4)	-0.8(4)
N1	12.6(5)	18.2(6)	11.5(5)	-0.8(4)	0.8(4)	-0.4(4)
C6	13.8(6)	19.2(7)	18.9(6)	-1.3(5)	-2.2(5)	0.1(4)
C4	13.3(6)	16.8(6)	15.0(6)	1.4(5)	1.2(4)	1.0(4)
C2	13.6(6)	16.2(6)	13.7(6)	-0.6(5)	0.7(4)	-0.5(4)
C7	14.8(6)	25.7(7)	21.9(7)	0.7(5)	0.0(5)	-3.5(5)
C5	13.9(6)	29.5(7)	18.1(6)	0.9(5)	-0.6(5)	-2.4(5)
C1	13.6(6)	15.1(6)	10.9(6)	0.5(4)	0.6(4)	1.2(4)
C3	15.6(6)	20.5(7)	12.2(6)	-0.2(5)	-0.6(4)	-0.7(5)

Table 4 Bond Lengths for **15**.

**Atom**	**Atom**	**Length/Å**	**Atom**	**Atom**	**Length/Å**
S1	C1	1.7156(14)	N2	C1	1.3714(16)
S1	C3	1.7289(13)	N1	C2	1.3625(16)
O1	C4	1.2193(16)	N1	C1	1.3111(17)
O2	C6	1.3618(15)	C6	C7	1.4881(18)
O2	C2	1.3790(15)	C4	C5	1.4977(17)
O3	C6	1.1932(16)	C2	C3	1.3500(17)
N2	C4	1.3649(16)			

Table 5 Bond Angles for **15**.

**Atom**	**Atom**	**Atom**	**Angle/°**	**Atom**	**Atom**	**Atom**	**Angle/°**
C1	S1	C3	89.24(6)	N2	C4	C5	115.02(11)
C6	O2	C2	122.05(10)	N1	C2	O2	111.27(10)
C4	N2	C1	124.12(11)	C3	C2	O2	131.26(11)

C1	N1	C2	109.38(11)	C3	C2	N1	117.41(11)
O2	C6	C7	109.86(11)	N2	C1	S1	124.72(10)
O3	C6	O2	123.40(12)	N1	C1	S1	115.30(10)
O3	C6	C7	126.73(12)	N1	C1	N2	119.98(12)
O1	C4	N2	121.39(12)	C2	C3	S1	108.64(9)
O1	C4	C5	123.59(11)				

Table 6 Hydrogen Atom Coordinates (Å×10^4) and Isotropic Displacement Parameters (Å^2×10^3) for **15**.

**Atom**	***x***	***y***	***z***	**U(eq)**
H2	4414.91	1837.55	2900.05	17
H7A	7230.56	4788.6	3168.81	31
H7B	7647.5	5306.63	4323.17	31
H7C	7153.04	6758.01	3840.74	31
H5A	3221.94	-528.37	3464.69	31
H5B	3546.43	744.43	2451.54	31
H5C	3038.53	1617.02	3326.56	31
H3	5874.58	3555.2	6575.21	19

Compound 19 CCDC 2330480

Table 1 Crystal data and structure refinement for **19**.

Identification code	AVS_30_11_23_dry_auto
Empirical formula	$C_5H_5ClN_2OS$
Formula weight	176.62
Temperature/K	100.01(10)
Crystal system	triclinic
Space group	P-1
a/Å	5.0499(2)
b/Å	7.4591(3)
c/Å	9.4964(3)
α/°	90.462(3)
β/°	91.106(3)
γ/°	108.081(4)
Volume/Å^3	339.94(2)
Z	2
ρ_{calc}g/cm^3	1.725
μ/mm^{-1}	0.790
F(000)	180.0
Crystal size/mm^3	0.1 × 0.02 × 0.02
Radiation	Mo Kα (λ = 0.71073)

2Θ range for data collection/°	4.29 to 60.426
Index ranges	-6 ≤ h ≤ 6, -10 ≤ k ≤ 10, -13 ≤ l ≤ 13
Reflections collected	7149
Independent reflections	1682 [R_{int} = 0.0437, R_{sigma} = 0.0246]
Data/restraints/parameters	1682/0/92
Goodness-of-fit on F^2	1.067
Final R indexes [I>=2σ (I)]	R_1 = 0.0251, wR_2 = 0.0691
Final R indexes [all data]	R_1 = 0.0264, wR_2 = 0.0701
Largest diff. peak/hole / e $Å^{-3}$	0.46/-0.33

Table 2 Fractional Atomic Coordinates (×10^4) and Equivalent Isotropic Displacement Parameters ($Å^2$×10^3) for **19**. U_{eq} is defined as 1/3 of of the trace of the orthogonalised U_{IJ} tensor.

**Atom**	***x***	***y***	***Z***	**U(eq)**
S1	2669.8(6)	1879.7(4)	5997.4(3)	11.08(10)
Cl1	1273.9(6)	2319.4(4)	9030.3(3)	14.75(10)
O1	2940.6(19)	1076.5(13)	3269.9(10)	18.4(2)
N2	6930(2)	3117.6(14)	4156.9(10)	11.1(2)
N1	7566(2)	4298.3(14)	6464.7(11)	12.3(2)
C3	5982(2)	3202.7(16)	5493.4(12)	10.0(2)
C2	6167(3)	4112.6(17)	7714.4(13)	12.9(2)
C4	5350(3)	2019.3(16)	3096.5(12)	12.2(2)
C5	6863(3)	2085.8(18)	1737.5(13)	15.4(2)
C1	3554(3)	2884.7(16)	7664.4(12)	12.1(2)

Table 3 Anisotropic Displacement Parameters ($Å^2$×10^3) for **19**. The Anisotropic displacement factor exponent takes the form: $-2\pi^2[h^2a^{*2}U_{11}+2hka^*b^*U_{12}+\ldots]$.

**Atom**	$\mathbf{U_{11}}$	$\mathbf{U_{22}}$	$\mathbf{U_{33}}$	$\mathbf{U_{23}}$	$\mathbf{U_{13}}$	$\mathbf{U_{12}}$
S1	7.56(16)	11.78(16)	11.48(16)	-0.33(11)	2.27(11)	-0.64(11)
Cl1	12.32(17)	18.38(17)	11.78(16)	0.46(11)	5.50(11)	1.87(12)
O1	10.9(4)	20.2(5)	18.7(5)	-4.6(4)	1.2(3)	-2.9(4)
N2	7.1(5)	12.5(5)	11.3(5)	-0.4(4)	2.3(4)	-0.6(4)
N1	9.2(5)	13.1(5)	12.7(5)	-0.6(4)	1.4(4)	1.0(4)
C3	6.8(5)	10.3(5)	12.3(5)	1.0(4)	1.5(4)	1.5(4)
C2	11.4(6)	14.9(5)	12.1(5)	-0.9(4)	0.7(4)	3.4(4)
C4	11.3(6)	11.4(5)	13.2(5)	-0.9(4)	0.8(4)	2.4(4)
C5	14.7(6)	17.4(6)	12.3(5)	-2.5(4)	2.2(4)	2.5(5)
C1	11.6(5)	13.9(5)	10.9(5)	0.5(4)	2.6(4)	4.1(4)

Table 4 Bond Lengths for **19**.

Atom	Atom	Length/Å	Atom	Atom	Length/Å
S1	C3	1.7362(12)	N2	C4	1.3681(15)
S1	C1	1.7359(12)	N1	C3	1.3086(15)
Cl1	C1	1.7180(12)	N1	C2	1.3789(15)
O1	C4	1.2169(16)	C2	C1	1.3522(18)
N2	C3	1.3730(15)	C4	C5	1.5058(17)

Table 5 Bond Angles for **19**.

Atom	Atom	Atom	Angle/°	Atom	Atom	Atom	Angle/°
C1	S1	C3	87.21(6)	O1	C4	N2	121.57(11)
C4	N2	C3	123.44(11)	O1	C4	C5	124.27(11)
C3	N1	C2	109.99(10)	N2	C4	C5	114.15(10)
N2	C3	S1	122.68(9)	Cl1	C1	S1	121.79(7)
N1	C3	S1	116.33(9)	C2	C1	S1	111.61(9)
N1	C3	N2	121.00(11)	C2	C1	Cl1	126.60(10)
C1	C2	N1	114.85(11)				

Table 6 Hydrogen Atom Coordinates (Å×10^4) and Isotropic Displacement Parameters (Å^2×10^3) for **19**.

Atom	*x*	*y*	*z*	U(eq)
H2	8643.35	3805.52	3972.81	13
H2A	6975.64	4794.88	8546.6	16
H5A	5652.16	1198.5	1053.63	23
H5B	8557.7	1737.16	1911.78	23
H5C	7365.39	3365.14	1362.57	23
